# Propolis Flavonoids: Current Evidence in Animal Models and Future Promise for the Treatment of Ulcerative Colitis

**DOI:** 10.3390/ijms27209048

**Published:** 2026-10-12

**Authors:** Karla Mariela Hernández-Sánchez, Oscar Nieto-Yañez, Claudia Rebeca Rivera-Yañez, Alexander Pedroza-González, Laura Francisco-Cruz, Claudia Tzasna Hernández-Delgado, Axel Rodrigo Molina-Gallardo, Nancy Aline Hernández-Rosas, Porfirio Alonso Ruiz-Hurtado, Cecilia Carlota Barrera-Ortega, Nelly Rivera-Yañez

**Affiliations:** 1 Laboratorio de Química de Productos Naturales, Departamento de Química Orgánica, Escuela Nacional de Ciencias Biológicas, Instituto Politécnico Nacional, Prol. de Carpio y Plan de Ayala, Miguel Hidalgo, Ciudad de México 11340, Mexico; kmhernandez@ipn.mx; 2 Laboratory in Nano and Dental Biomaterials, Facultad de Estudios Superiores Iztacala, Universidad Nacional Autónoma de México (UNAM), Tlalnepantla 54090, Mexico; oscar.nieto@iztacala.unam.mx (O.N.-Y.); lauurefcr@gmail.com (L.F.-C.); 3 Carrera de Médico Cirujano, Facultad de Estudios Superiores Iztacala, Universidad Nacional Autónoma de México (UNAM), Tlalnepantla 54090, Mexico; drarrivera@unam.mx (C.R.R.-Y.); alexander_pg@comunidad.unam.mx (A.P.-G.); 4 Laboratorio de Inmunología, Unidad de Morfología y Función, Facultad de Estudios Superiores Iztacala, Universidad Nacional Autónoma de México (UNAM), Tlalnepantla 54090, Mexico; 5 Laboratorio de Bioactividad de Productos Naturales, Unidad de Biotecnología y Prototipos (UBIPRO), Facultad de Estudios Superiores Iztacala, Universidad Nacional Autónoma de México (UNAM), Tlalnepantla 54090, Mexico; tzasna@unam.mx (C.T.H.-D.); axel.molina@iztacala.unam.mx (A.R.M.-G.); 6 Laboratorio de Toxicología Molecular, Departamento de Farmacia, Escuela Nacional de Ciencias Biológicas, Instituto Politécnico Nacional, Av. Wilfrido Massieu, Esq. Manuel L. Stampa s/n, Gustavo A. Madero, Ciudad de México 07738, Mexico; alinehdzro@gmail.com (N.A.H.-R.); pruizhu@ipn.mx (P.A.R.-H.); 7 Laboratorio de Toxicología de Productos Naturales, Departamento de Farmacia, Escuela Nacional de Ciencias Biológicas, Instituto Politécnico Nacional, Av. Wilfrido Massieu, Gustavo A. Madero, Ciudad de México 07738, Mexico; 8 División de Investigación y Posgrado, Facultad de Estudios Superiores Iztacala, Universidad Nacional Autónoma de México (UNAM), Tlalnepantla 54090, Mexico

**Keywords:** bioactive compounds, flavonoids, anti-inflammatory activity, cytokines, tight junction proteins, antioxidant activity, signaling pathways

## Abstract

Ulcerative colitis is a chronic, immune-mediated inflammatory bowel disease with a rising global incidence and a considerable economic burden. Conventional treatments, such as steroidal anti-inflammatory drugs and immunosuppressants, are often associated with adverse effects and high costs. In this context, flavonoids—such as those identified in the chemical composition of propolis—are emerging as highly promising therapeutic alternatives. This review highlights how diverse classes of flavonoids, including flavones, flavonols, flavanones, isoflavones, and flavan-3-ols, mitigate colitis through multiple interconnected mechanisms. Their main actions include reducing inflammation by decreasing the levels of proinflammatory cytokines and modulating key signaling pathways; exerting potent antioxidant activity; and improving intestinal barrier integrity by increasing tight junction proteins and mucin production. Numerous studies reporting significant improvements in clinical parameters such as body weight, colon length, and the disease activity index are discussed. Innovation in formulations, for example, the use of nanoparticles, is crucial to improve the solubility, stability, and bioavailability of flavonoids, allowing for their targeted delivery to the inflamed colon. In summary, flavonoids are promising candidates for the development of complementary or alternative treatments for ulcerative colitis. Although further clinical evidence is needed to establish their safety and conditions for use in humans, the available preclinical results are promising.

## 1. Introduction

### 1.1. Ulcerative Colitis

Ulcerative colitis (UC) is classified as an inflammatory bowel disease. It is a chronic immune-mediated inflammatory disorder of the colon and rectum characterized by persistent or recurrent abdominal pain, diarrhea, mucus, and bloody stools. The pathogenesis of UC is complex, involving genetic and environmental factors as well as an abnormal immune response [1]. Regarding its pathophysiology, more than 200 genetic loci, as well as environmental exposures, have been linked to pathogens that cause dysbiosis and a deficient immune response against the microbiome. These affect the epithelial barrier, resulting in the initiation and perpetuation of inflammation [2].

Over the past two decades, UC has gained increasing prominence as a global public health issue, driven by complex and escalating changes in its epidemiological patterns [1]. Notably, this situation is particularly concerning in countries with the highest UC prevalence rates, such as the UK (570 per 100,000), Denmark (523 per 100,000), and Norway (500 per 100,000). At the same time, countries in Asia and Latin America have also registered significant increases in the prevalence of UC [3]. The public health burden of UC extends to the economic sector, and it has been estimated that the annual expenditure associated with this disease ranges from EUR 12.5 to 29.1 billion in Europe and USD 8.1 to 14.9 billion in the United States [4].

Currently, there is no cure for ulcerative colitis, with first-line treatment for mild-to-moderate cases focusing primarily on 5-aminosalicylic acid (5-ASA) agents, including sulfasalazine, olsalazine, balsalazide, and mesalazine (also known as mesalamine) [5]. New therapeutic alternatives are emerging that offer both promising efficacy and fewer undesirable side effects, such as bone marrow stem cell transplantation, biological agents (e.g., vedolizumab and ustekinumab), and fecal microbiota transplantation [1]. Nevertheless, these treatments are still costly and have limited distribution, making them difficult to access in developing countries and marginalized communities.

Beehive products have been consumed for millennia, and remain relevant today due to the numerous beneficial effects that have been attributed to them. Regarding colitis, the various biological properties reported by the scientific community make such products promising candidates in the search for easily accessible and affordable therapeutic options. Given the scarcity of studies on the activity of propolis against UC, this review focuses on the flavonoids identified in the chemical composition of propolis and their effects against UC reported in preclinical studies, highlighting their significant therapeutic potential for this disease.

### 1.2. Propolis with Activity Against Ulcerative Colitis

Propolis is a natural resinous product which is produced by bees from various botanical materials, such as resins and floral exudates, that are subsequently mixed with beeswax and enzymes secreted via their salivary glands [6]. Its chemical composition is highly variable and depends on factors such as the botanical origin, soil and climatic conditions, season of collection, and the bee species [7,8]. This diversity of raw materials gives rise to a complex composition, comprising more than 600 chemical compounds including esters, flavonoids, terpenes, aldehydes, aromatic alcohols, fatty acids, stilbenes, and steroids [9,10]. Among these components, flavonoids, phenolic acids, and terpenoids stand out as the primary compounds with biological activity [10,11], contributing to the potential of propolis as a natural substance for the treatment of UC.

Various studies have demonstrated that propolis from different sources exhibits protective effects against experimental colitis in mice. Particularly, extracts derived from Brazilian and Korean green propolis have shown the ability to reduce disease severity and the disease activity index (DAI), prevent weight loss and colon shortening, and decrease histopathological damage. The authors of [12] observed similar effects with Brazilian green propolis (23.3 mg/g of diet) in a 2,4,6-trinitrobenzene sulfonic acid (TNBS)-induced colitis model in mice, while other authors [13,14] have reported similar results in dextran sulfate sodium (DSS)-induced colitis mouse models when using Brazilian green propolis (300 mg/kg or a 0.3% diet). Furthermore, Mariano et al. [13] demonstrated improved mucin preservation and increased antioxidant defenses, in terms of the levels of enzymes such as superoxide dismutase (SOD), while Shimizu and Suzuki [14] showed the preservation of tight junction proteins (including ZO-1, ZO-2, occludin, JAM-A, claudin-3, and claudin-4) and reductions in proinflammatory cytokines (such as TNF-α, IL-1β, and IL-17A). Similarly, another study [15] reported that Korean propolis (administered at dilutions of 1:1000 and 1:500) reduced histological injury, preserved goblet cells, and decreased lymphocyte infiltration and COX-2 expression. Taken together, these findings indicate that propolis samples from Brazil and Korea share anti-inflammatory and intestinal-mucosa-protective effects, which are associated with reduced tissue damage and the maintenance of intestinal barrier integrity.

Various studies have demonstrated that propolis of Chinese and Brazilian origin exerts protective effects in experimental rat models of colitis, primarily by reducing inflammation and oxidative stress, as well as by preserving intestinal mucosal integrity. Some authors [16,17] have observed that both Chinese propolis and Brazilian green propolis, administered at 300 mg/kg or as a 0.3% diet, reduced the DAI, colon shortening, histological damage, and epithelial cell apoptosis in rats with DSS-induced colitis. Furthermore, both extracts decreased the expression of the proinflammatory cytokines IL-1β and IL-6 and the chemokine MCP-1, as well as lipid peroxidation, evidencing the anti-inflammatory and antioxidant effects of both types of propolis.

Other authors [18] found that water-soluble Chinese propolis, administered at 100 or 200 mg/kg, improved clinical signs of colitis, reduced the DAI and macroscopic and histological damage, and prevented colon shortening in rats with DSS-induced colitis. This treatment also decreased myeloperoxidase (MPO) activity and the levels of TNF-α, IL-6, and IL-9, indicating reduced neutrophil infiltration and activation. Concurrently, it increased the activity of the antioxidant enzymes SOD and glutathione peroxidase (GPx) and reduced the levels of reactive oxygen species (ROS) and malondialdehyde (MDA), suggesting a dose-dependent protective effect associated with the attenuation of inflammation and oxidative stress.

In addition, the protective effects of Brazilian brown propolis in rats with TNBS-induced colitis have also been demonstrated. In 2024, Cury et al. [19] reported that the administration of 100 or 300 mg/kg of the hydroalcoholic extract reduced macroscopic lesions and histological damage, promoted mucosal preservation, and increased the mucin content. Both doses decreased lipid peroxidation and MDA levels, while the 300 mg/kg dose prevented colon shortening and restored reduced glutathione (GSH) levels. Furthermore, a decrease in MPO activity was observed, which was associated with reduced neutrophil migration.

Similarly, other authors [20] have evaluated the effects of a hydroalcoholic extract of Brazilian red propolis in rats with acetic-acid-induced colitis. Treatment with 10 mg/kg reduced macroscopic and histological damage, inflammation, and iNOS expression, while decreasing MPO activity. The authors suggested that Brazilian red propolis exhibited protective and anti-inflammatory effects, primarily associated with reduced neutrophil infiltration and iNOS inhibition.

In 2024, the authors of [21] evaluated the effects of water-soluble Chinese propolis in rats with DSS-induced colitis to explore potential signaling pathways that could partially explain the activities of the propolis against colitis. Treatment with Chinese propolis at 100 or 200 mg/kg significantly reduced the disease activity index and improved mucosal integrity and the colonic architecture. Furthermore, it decreased the expression of PKC (Protein Kinase C), TRPV1 (Transient Receptor Potential Cation Channel Subfamily V Member 1), CGRP (Calcitonin Gene-Related Peptide), and SP (Substance P), as well as their co-localization in colonic tissue. Taken together, their results suggest that Chinese propolis exerts a protective effect against colitis by inhibiting the PKC–TRPV1–CGRP/SP pathway, thereby reducing intestinal inflammation and visceral hypersensitivity.

Furthermore, it has recently been reported that various propolis formulations exhibit enhanced bioactivity; for instance, in 2025, Franchin et al. [22] observed that the oral administration of alginate microcapsules containing an ethanolic extract of Brazilian green propolis (at a dose of 300 mg/kg) in a murine model of DSS-induced acute colitis prevented body weight loss and colon shortening, reduced histological lesions in the colonic mucosa by 86.1% (compared to 54.9% with non-encapsulated propolis), and significantly lowered TNF-α, IL-6, and IL-1β levels in colonic tissue; however, it should be noted that there were no significant differences regarding the latter compared to non-encapsulated propolis. Consequently, the authors attributed the enhanced protective effect of the propolis to microencapsulation via ionotropic gelation—a process that protects bioactive compounds during the gastric phase and promotes their sustained release in the intestine, potentially increasing the therapeutic efficacy of the propolis extract.

The available evidence indicates that propolis from different geographic origins, particularly Brazil, China, and Korea, exhibits significant protective effects against experimental colitis. These effects are mainly associated with the attenuation of intestinal inflammation and oxidative stress, preservation of the intestinal mucosal barrier, reduction of histopathological damage, and maintenance of colonic architecture. Although these biological activities are shared among different types of propolis, variations in their efficacy may be influenced by their geographical origin, chemical composition, extraction method, dose, and formulation. Therefore, propolis represents a promising source of bioactive compounds for the prevention or management of intestinal inflammatory disorders, although further studies are necessary to clarify its mechanisms of action and therapeutic potential.

### 1.3. Propolis Flavonoids

Flavonoids are plant secondary metabolites belonging to the group of phenolic compounds. These are widely distributed in the plant kingdom, and are present in many foods such as vegetables, herbs, spices, legumes, and fruits; they are also abundant in bee products, as bees incorporate them from the raw materials that they collect [23,24]. Flavonoids have various biological activities in plants, such as protection against ultraviolet light, pigmentation, and the stimulation of nitrogen-fixing nodules, and also play essential roles in defense against pathogens [25,26]. While numerous studies have reported the anti-inflammatory properties of propolis, few have focused on colitis. We consider that the flavonoids found in this product could be promising candidates in the search for new strategies to complement the existing treatment modalities for this disease, given that propolis contains a complex mixture of flavonoids belonging to different structural subclasses, including flavones, flavonols, flavanones, isoflavones, and flavan-3-ols [7,8]. Therefore, we review and analyze the most relevant studies from the past fifteen years regarding preclinical research on flavonoids in experimental models of colitis.

Flavonoids have a characteristic core structure consisting of two benzene rings (A and B) connected via a 3-carbon bridge (heterocyclic ring containing oxygen, C) [27,28]. Their biosynthesis involves two metabolic pathways: the acetate pathway for ring A, and the shikimate pathway for rings B and C. As all flavonoids are synthesized in this way, they share a basic structure [29,30]. Based on the positions of functional (e.g., hydroxyl) groups on the different rings of this structure, flavonoids can be classified into flavones, flavonols, flavan-3-ols, isoflavones, flavanones, and anthocyanidins [23,27]. Table 1 shows some examples of compounds belonging to each group of flavonoids.

There is clinical and epidemiological evidence that consuming flavonoids helps to mitigate chronic diseases, including cardiovascular disease, diabetes, obesity, Alzheimer’s disease, inflammatory bowel disease, and even colon cancer [31,32,33,34,35], and the antioxidant and anti-inflammatory properties of flavonoids have been widely described. Consequently, they represent promising candidates for use in the treatment of various diseases associated with inflammation [23,26,36].

### 1.4. Flavonoid Metabolism

In plants, most flavonoids occur as glycoside conjugates. Once flavonoids have been consumed (e.g., as part of bee products), their absorption usually occurs in the small intestine. At this site, the flavonoids are converted into aglycones through a hydrolytic reaction carried out by an enzyme known as lactase-phlorizin hydrolase (LPH), and the released aglycone can enter epithelial cells via passive diffusion [37,38,39]. An alternative pathway has also been proposed, where ingested flavonoids are taken into epithelial cells by active sodium-dependent glucose transporter 1 (SGLT1) and glucose transporter 2 (GLUT2) [39,40,41]. The next step is performed by the enzymes sulfotransferases (SULT), uridine-50-diphosphate glucuronosyltransferases (UGTs), and catechol-O-methyltransferases (COMT), which transform aglycones into sulfates, glucuronides, and/or methylated metabolites that then pass into the bloodstream to be distributed systemically. Many of these metabolites may undergo phase II metabolism. It should be clarified that not all the metabolites generated reach the circulation, as some are returned to the intestinal cavity through the action of proteins such as P-glycoprotein (P-gp), multi-drug resistance-related protein (MRP), and breast cancer resistance protein (BCRP) [42,43]. In the intestine, flavonoids (aglycones or glucuronides) undergo metabolic processes that are closely linked to the microbiota—mainly bacteria that produce hydrolytic enzymes, which generate aglycones and promote the production of smaller molecules, such as phenolic acids and hydroxycinnamates, which can be absorbed, undergo further metabolism in the liver, and are excreted in urine. Research on the metabolism and pharmacokinetics of flavonoids in the human body continues to focus on optimizing their use and consumption [41,44].

As mentioned above, the flavonoids that are present in propolis can have diverse systemic effects. While more than 7000 flavonoids with myriad biological properties have been described, we compile the most relevant information from research conducted over the last 15 years in this study, focusing on compounds which have significant potential for the treatment and mitigation of UC.

## 2. Flavones with Activity Against Ulcerative Colitis

A study has reported that, in rats with acetic-acid-induced colitis, the oral administration of apigenin (3 mg/kg) daily for seven days improved body weight and colon length, increased GSH levels, and reduced the DAI, MDA, Mucosal Targeting Cell Adhesion Molecule-1 (MAdCAM-1), total nitrite/nitrate (NOx), and IL-1β levels, as well as MPO activity in the colon. Therefore, the authors suggested that this flavonoid could be beneficial for the treatment of colitis [45] (Table 2).

Eun et al. observed that tangeretin treatment (10 or 20 mg/kg, via oral gavage for 3 days) inhibited MPO activity and MAPK and NF-κB activation in the colon of TNBS-induced ulcerative colitis mice. Additionally, it increased the expression of ZO-1, claudin-1, and occludin (tight junction proteins); suppressed the differentiation of Th17 and Th1 cells; and decreased the expression of TNF-α, IL-12, IL-17, IFN-γ, Retinoid-related orphan receptor gamma t (RORγt), and T-box expressed in T cells (T-bet). Furthermore, tangeretin induced the differentiation of regulatory T (Treg) cells and the expression of IL-10 and Foxp3. These results suggest that this compound could ameliorate colitis by inhibiting TNF-α and IL-12 expression and NF-κB activation [46].

Another study in TNBS-induced ulcerative colitis rats demonstrated that baicalin treatment (100 mg/kg twice a day for 7 days) down-regulated the quantity of macrophages and the expression of macrophage migration inhibitory factor (MIF), MCP-1, and MIP-3α, suggesting that this compound may be a complementary option in the treatment of colitis [47]. Zhu et al. described the effects of baicalin on the Th17/Treg equilibrium in TNBS-induced ulcerative colitis rats. Baicalin administration (25, 50, or 100 mg/kg intragastrically every 2 days for 14 days) decreased the DAI in a dose-dependent manner, while also mitigating mucosal damage and increasing Mucin 2 (MUC2), occludin, and ZO-1 protein expression. In addition, baicalin decreased MDA and ROS levels and increased SOD and GSH levels in colonic tissue; furthermore, it mitigated the up-regulation of the Th17/Treg ratio, resulting in an improvement in inflammation in the colon. The authors concluded that baicalin likely protects against colitis—at least in part—through modulation of the Th17/Treg equilibrium, and that it could be a promising candidate for the treatment of colitis [48]. Similarly, the effect of baicalin on the equilibrium of Th17 and Treg cells has been studied in TNBS-induced ulcerative colitis rats. Baicalin administration (10 mL/kg orally twice a day for 7 days) decreased both the macroscopic and microscopic scores and the DAI; ameliorated shortening of the colon and weight loss; and reduced IL-1β and TNF-α levels, IFN-γ and IL-12 (Th1-related cytokines) expression, and MPO activity. In addition, baicalin down-regulated the quantity of Th17 cells and the levels of IL-6, IL-17 (Th17-related cytokines), and RORγt, while increasing the number of Treg cells and the levels of Foxp3, IL-10, and TGF-β (Treg-related cytokines). These results further suggest that the anti-inflammatory activity of baicalin in models of colitis could be related to regulation of the equilibrium between Treg and Th17 cells [49]. Zhu et al. observed that administration of baicalin (100 mg/kg via oral gavage for 14 days) in TNBS-induced ulcerative colitis rats decreased IL-1β, TNF-α, and IL-6 secretion; raised IL-10 levels; and stimulated ZO-1 protein expression, while suppressing the PI3K/AKT signaling pathway. This suggests that baicalin likely ameliorated colitis in this model through suppression of PI3K/AKT activation [50]. One investigation reported that baicalin treatment (30, 60, or 90 mg/kg intragastrically for 28 days) increased GSH, CAT, and SOD activities and reduced TNF-α, IL-1β, MPO, MDA, and prostaglandin E_2_ (PGE_2_) contents in the colonic tissue of TNBS-induced ulcerative colitis rats. In addition, this compound reduced cyt-c, cleaved-caspase9, cleaved-caspase3, NF-κB (p-65), p-IκBα/IκBα, and p-IKKβ/IKKβ expression. These results suggest that baicalin’s colitis-modulating activity is mediated by the IKK/IκB/NF-κB signaling pathway and proteins associated with apoptosis [51]. Another study found that administration of baicalin (5, 2.5, or 1.25 mg/mL orally for 15 days) attenuated TNBS-induced ulcerative colitis in rats. Furthermore, this compound decreased IL-6, TNF-α, and IL-1β production, which was linked to a reduction in mucosal TLR4 protein expression, and reduced p-NF-κB (p65) protein expression, which was linked to a decrease in the p-IκB protein. These results suggest that baicalin has a suppressive effect on TLR4/NF-κB pathway activation in this colitis model [52].

Cheng et al. reported that the administration of a tangeretin-containing diet (0.04% or 0.08%) for 37 days mitigated damage in DSS-induced ulcerative colitis mice, as evidenced by changes in colon tissue, the DAI, and colon length shortening. Furthermore, this compound down-regulated IL-6, IL-1β, and TNF-α in the colon and ameliorated ZO-1 and claudin-1 expression. These results suggest that tangeretin could be a promising candidate for the prevention and/or treatment of colitis [53] (Table 3).

Ren et al. found that acacetin treatment (50 or 150 mg/kg via oral gavage, starting on day 9 in DSS-induced ulcerative colitis mice) decreased diarrhea, colon shortening, histological damage, body weight loss, and inflammatory infiltration. Furthermore, this compound reduced the infiltration of macrophages expressing F4/80 and decreased iNOS, COX-2, TNF-α, IL-1β, and IL-6 mRNA levels in colonic tissue. These results suggest that acacetin may mitigate colitis, possibly by suppressing inflammation and decreasing the production of proinflammatory mediators [54].

One study mentioned that pre-administration of apigenin (25 µg intraperitoneally, twice a week for 3 weeks) ameliorated shortening of the colon length, body weight loss, the histological score, and the IL-1β level in DSS-induced ulcerative colitis mice. This compound also regulated the NLRP6 signaling pathway, and the authors concluded that apigenin could be considered as a candidate for the treatment of colitis [55]. In another study, it was reported that the intragastric administration of apigenin (150 or 250 mg/kg) once daily for 21 days to mice with DSS-induced colitis improved weight loss; decreased the DAI score and levels of IL-1β, IL-6, and TNF-α; and increased IL-10 levels, MPO activity, and the expression of occludin, claudin-1, and ZO-1, with a dose-dependent effect observed for all parameters evaluated. The authors concluded that this flavonoid could be a promising candidate for the treatment of colitis [56]. Another investigation assessed the protective activities of pre-treatment for 30 days with an apigenin-containing diet (0.3%) in DSS-induced ulcerative colitis mice. Apigenin pre-treatment reduced the macroscopic and microscopic effects of colitis, down-regulated COX-2 and iNOS expression in the colon, and reduced serum matrix metalloproteinase (MMP-3) levels. In addition, IL-18 and IL-1β levels were reduced as a result of the modulation of cleaved caspase-1 and caspase-11 enzymes, demonstrating that the anti-inflammatory effect of this compound is associated with suppression of canonical and non-canonical NLRP3 inflammasome pathways. The authors concluded that a diet including apigenin could be included as part of alternative or preventive treatments for colitis [57].

A study reported that the oral administration of baicalein daily for 10 days at a concentration of 40, 20, or 10 mg/kg in mice with DSS-induced colitis improved their body weight and colon length, reduced the DAI score, and increased colonic occludin and ZO-1 levels. It also positively modulated the aryl hydrocarbon (AhR)/IL-22 pathway in group 3 innate lymphoid cells (ILC3s). Notably, ILC3s found in the intestine produce the anti-inflammatory cytokine IL-22, whose function is influenced by the AhR receptor and is involved in protecting and repairing the intestinal barrier, which is relevant to inflammatory diseases such as ulcerative colitis [58]. Yao et al. observed that administration of baicalein (10, 20, or 40 mg/kg intragastrically for 10 days) in DSS-induced ulcerative colitis mice decreased their body weight loss in a dose-dependent manner, diarrhea, rectal bleeding, colonic ulceration, colon length shortening, and the number of CD11b^+^ cells in the colon. This compound also mitigated expression of the transcription factor RORγt; decreased TNF-α, IL-1β, and IL-6 levels in the colon and serum; and suppressed p-STAT3, sphingosine kinase-1 (SPHK1), nucleotide-binding oligomerization domain 2 (NOD2), and sphingosine 1-phosphate receptor 1 (S1PR1) expression in colonic tissue. The authors concluded that this compound ameliorated colitis, likely through suppression of S1P-STAT3 signaling, and thus could be considered as a promising candidate for use in the treatment of this disease [59].

One study reported that pre-treatment with baicalin (100 mg/kg intragastric for 10 days) reduced the DAI in DSS-induced ulcerative colitis mice, thus ameliorating the severity of the disease. Furthermore, this compound increased the mRNA expression of IL-10 and decreased those of IL-23 and TNF-α, suggesting that baicalin has therapeutic potential for the treatment of diseases such as colitis [60]. In 2024, Zhang et al. reported that the administration of a magnesium baicalin preparation (200 or 100 mg/kg) via intraperitoneal injection daily for 9 days showed a dose-dependent effect in rats with DSS-induced colitis, with the highest concentration showing the best results. In particular, the preparation improved body weight and colon length; reduced the DAI and the levels of IL-1β, IL-6, TNF-α, and MDA; and increased the levels of IL-10, SOD, claudin-1, occludin, and ZO-1. These results suggest that the magnesium baicalin preparation improves the water solubility of this flavonoid, as well as its therapeutic effectiveness against colitis in rats, indicating its significant potential for assessment as a treatment option in further clinical studies for colitis [61]. Another study reported that the oral administration of baicalin (100, 50, or 25 mg/kg) daily for 7 days in mice with DSS-induced colitis improved their body weight and colon length; reduced the DAI score and serum levels of TNF-α, IL-1β, and IL-6; increased the levels of E-cadherin, ZO-1, claudin-5, claudin-4, IL-18, and MUC2, as well as NLRP6 production; and reduced caspase-1 production in colonic tissue. Furthermore, the effect of baicalin was dose-dependent, with the highest dose tested showing the best results. Therefore, the authors suggest that baicalin may alleviate colitis through activation of the NLRP6 inflammasome, leading to improvement in intestinal mucosal barrier function and a reduction in inflammation [62]. In 2024, Jia et al. reported that the oral administration of a complex of L-arginine with baicalin (100 mg/kg) daily for 7 days improved body weight and colon length; reduced the levels of TNF-α, IL-1β, IL-6, and MPO; and increased the levels of ZO-1 and IL-10 in rats with DSS-induced colitis. They concluded that the formulation of the complex with this flavonoid improves its solubility and bioavailability and enhances its therapeutic effect on colitis in the tested animal model, making it a potential complementary alternative for consideration in future clinical applications [63].

Over the past 5 years, research on the effects of luteolin on colitis has increased considerably, with only one study from 2013 [64] and another from 2016 [65], while all other reports mentioned here were published from 2020 onward (Table 4). The studies conducted in 2025 by Yang et al. [66] and in 2024 by Bi et al. [67] reported that daily administration (for 14 days) of luteolin (25 mg/kg, orally) or luteolin-loaded rapid self-assembling hydrogels (5 mg/kg, contained in the water consumed by mice), respectively, in mice with colitis induced by DSS improved their body weight and colon length, reduced the DAI score, and—both systemically (serum) and locally (colonic tissue)—increased the expression of claudin-1, occludin, and ZO-1; decreased the levels of IL-1β, IL-6, and TNF-α; and increased the production of IL-10. Therefore, both groups suggested that this flavonoid may be considered as a potential novel nutritional supplement for the treatment of colitis [66,67]. In another study, luteolin treatment (20 or 50 mg/kg, administered intragastrically for 18 days) improved the histological score and colon length shortening in DSS-induced ulcerative colitis mice. It also suppressed IFN-γ-producing CD4^+^ T cells, macrophage infiltration, and COX-2, IFN-γ, TNF-α, IL-1β, and IL-6 mRNA expression in the colon [64]. Another study demonstrated that luteolin treatment (20 or 50 mg/kg orally for 7 days) mitigated colon histological damage, the DAI, and colon shortening in DSS-induced ulcerative colitis mice. Furthermore, this compound reduced IL-6, TNF-α, and iNOS expression and MDA content; increased CAT and SOD activities; and increased the levels of Nuclear factor erythroid 2 (NF-E2)-related factor 2 (Nrf2) and its downstream targets NAD(P)H: quinone oxidoreductase 1 (NQO1) and heme oxygenase-1 (HO-1) in the colon. These results indicate that this compound could inhibit ulcerative colitis through the Nrf2 signaling pathway [65]. A study reported that the oral administration of luteolin nanoparticles (i.e., nanoparticles manufactured using a D-α-tocopherol polyethylene glycol succinate-β-poly(β-thioester) copolymer) daily for 7 days at a concentration of 20 mg/kg improved body weight and colon length; reduced the DAI score; increased the expression of claudin-1, occludin ZO-1, and GSH; and reduced colonic ROS and MPO levels in DSS-induced colitis mice. It also decreased the levels of IL-6, IL-2, IL-17A, IFN-γ, and TNF-α and increased IL-10 and IL-4 production in both serum and colonic tissue. Finally, it increased the numbers of Th2 and Treg cells while decreasing the numbers of Th17 and Th1 cells. Therefore, the authors concluded that nanoparticles loaded with this flavonoid show promise as a therapeutic agent for the treatment of ulcerative colitis [68]. Li et al. demonstrated that administration of luteolin (50 or 100 mg/kg for 7 days) attenuated body weight loss in a dose-dependent manner, colon length shortening, the DAI, lesion size, and tissue damage. Furthermore, this compound up-regulated ZO-1, occludin, claudin-1, and SHP-1 and down-regulated claudin-2, P-STAT-3, and STAT3 (mRNA levels and protein expression) in the colon, indicating that, through an increase in SHP-1 expression, luteolin can stimulate epithelial barrier function [69]. Zou et al. reported that luteolin treatment (10 or 20 mg/kg, intramuscular injection for 10 days) attenuated body weight loss and colon shortening, raised SOD levels, and reduced MDA levels in the serum and colon in DSS-induced ulcerative colitis mice. Furthermore, this compound reduced TNF-α, IL-1β, and IL-6 levels in the colon and serum; decreased HMGB1 protein and mRNA levels; and decreased the expression of HMGB1–TLR–NF-κB signaling pathway-related proteins in the colon. This suggests that luteolin ameliorated colitis, where the associated mechanism was potentially linked to modulation of the intestinal HMGB1–TLR–NF-κB signaling pathway [70]. In another study, it was reported that the intragastric administration of luteolin at different concentrations (50 or 25 mg/kg) daily for 14 days improved body weight and colon length; reduced the DAI score; increased the expression of occludin, ZO-1, MUC4, MUC2, and MUC1; and decreased the levels of IL-1β, IL-6, and TNF-α in the colon of mice with DSS-induced colitis. It also reduced the phosphorylation levels of IKKα/β, IκBα, and p65, thereby decreasing the activation of the NF-κB signaling pathway in colonic tissue. Therefore, the authors suggested that this flavonoid has therapeutic potential against colitis [71]. In 2025, Yang et al. reported that the oral administration of luteolin daily for 17 days at different concentrations (50, 25, or 12 mg/kg) improved body weight and colon length; reduced the DAI score; increased the expression of claudin-1, occludin, and ZO-1 in the colon; decreased the levels of IL-1β, IL-6, and TNF-α; and increased IL-10 production in both the colon and serum of mice with DSS-induced colitis. It also up-regulated the expression levels of CD206 and Arg1 (M2-type macrophage markers) and down-regulated the expression levels of iNOS (a marker of M1-type macrophages) in the colon, while also increasing activation of the AMPK–PPAR-γ signaling pathway in colonic tissue. Therefore, the authors suggested that this flavonoid could be considered as a supplement to treat ulcerative colitis [72]. Vukelic et al. showed that the administration of luteolin (50 or 100 mg/kg orally for 7 days) decreased body weight loss, the DAI, and colon shortening in a dose-dependent manner in DSS-induced ulcerative colitis mice. This compound also increased ERK1/2 phosphorylation and reduced p-p38 and p-JNK1/2 expression in colonic tissue, and reduced p-STAT3 and p-Akt protein expression in the colon in a dose-dependent manner. These results suggest that this compound has anti-autophagic, anti-apoptotic, and anti-inflammatory properties which are mediated by activation of the ERK signaling pathway; thus, it could be a promising agent for the alternative or complementary treatment of colitis [73]. In 2024, Magadán-Corpas et al. reported that the intraperitoneal administration of luteolin daily for 11 days at a concentration of 10 mg/kg of body weight in rats with DSS-induced colitis resulted in the complete absence of colonic ulceration, as well as reductions in the DAI score and the levels of IL-1β and IL-6. These results suggest that this flavonoid could function as a potential co-therapy or alternative for the treatment of ulcerative colitis [74].

In one investigation, the administration of luteolin (34.6 mg/kg via gastric gavage for 14 days) in DSS-induced ulcerative colitis rats reduced IL-23, IL-17, and NF-κB levels and raised PPAR-γ levels, thus decreasing inflammation and damage in colon tissue, indicating that this compound could ameliorate colitis [75]. Other researchers have reported that the intragastric administration of luteolin daily for 14 days at a concentration of 34.6 mg/kg in rats with DSS-induced colitis improved body weight and colon length; decreased C-reactive protein, TNF-α, IL-33, and IL-13 levels and MDA concentrations; and elevated serum SOD levels and total antioxidant capacity (T-AOC). They concluded that this flavonoid could be beneficial in the treatment of ulcerative colitis [76]. Another study reported that the administration of luteolin-loaded polymeric micelles (100 mg/kg) via the intragastric route daily for four weeks improved the body weight; reduced the DAI score and the levels of IL-1β, IL-6, TNF-α, PGE_2_, MDA, and C-reactive protein (CRP); and increased the levels of SOD and IL-10 in both the serum and colon tissue in rats with TNBS-induced colitis. Therefore, the authors concluded that luteolin micelles have better activity in this model of colitis due to the improved stability, bioavailability, and release profile of the luteolin-loaded micelles. In the same study, they even reported having tested luteolin alone at the same concentration, through which it was confirmed that the luteolin micelles achieved the best results in terms of all evaluated parameters. Therefore, this flavonoid formulation shows promise for the treatment of colitis, and alternatives to improve its stability and bioavailability should continue to be studied [77].

Overall, the findings summarized above indicate that flavones may exert protective effects in experimental models of ulcerative colitis through the modulation of interconnected, rather than isolated, molecular pathways. Oxidative stress represents an important link between these pathways, as excessive ROS may promote inflammatory signaling through NF-κB and MAPK, whereas the activation of Nrf2 and its antioxidant downstream targets (e.g., NQO1 and HO-1) may contribute to the restoration of redox homeostasis and attenuation of inflammation. Consistent with this concept, luteolin has been reported to activate the Nrf2/NQO1/HO-1 pathway [65], tangeretin and luteolin were shown to modulate MAPK signaling [46,73], and several studies reported the inhibition of NF-κB signaling following treatment with baicalin or luteolin [51,52,70,71]. These pathways may also converge on inflammasome activation, since oxidative and inflammatory signals can contribute to NLRP3 inflammasome activation and the subsequent maturation of IL-1β and IL-18. In agreement with this mechanism, apigenin has been reported to suppress canonical and non-canonical NLRP3 inflammasome pathways through the modulation of caspase-1 and caspase-11 [57]. Other signaling nodes described in this section may contribute to complementary mechanisms of protection; for example, baicalein was found to modulate the AhR/IL-22 pathway, which is associated with intestinal barrier protection [58], whereas the modulation of S1P/STAT3 signaling was associated with the anti-inflammatory effects of baicalein [59]. Therefore, the available evidence supports a model in which flavones may simultaneously influence oxidative stress, inflammatory signaling, inflammasome activity, immune responses, and epithelial barrier integrity. However, these pathways should not be interpreted as a single linear cascade, as the studies summarized here generally evaluated individual signaling components and did not establish direct causal interactions among all of these pathways. Moreover, although pathway modulation activities have been demonstrated for several flavones, the available in vivo evidence does not generally establish direct binding of these compounds to the corresponding molecular targets. Thus, some effects may reflect the direct modulation of signaling components, whereas others may occur indirectly, for example, through changes in ROS and the cellular redox status.

The literature provides evidence for the remarkable therapeutic potential of flavones such as apigenin, tangeretin, baicalin, baicalein, and acacetin against UC, with research describing associated several mechanisms; for example, they reduce inflammation by decreasing levels of proinflammatory cytokines (e.g., TNF-α, IL-1β, and IL-6), inhibiting key pathways such as NF-κB and MAPK, and modulating the immune response (e.g., the Th17/Treg balance). They also exhibit antioxidant activity, which is mediated by increases in GSH and SOD and reductions in markers of oxidative damage such as MDA and ROS. Another important aspect is their ability to improve intestinal barrier integrity by increasing the expression of tight junction proteins such as ZO-1, occludin, and claudin-1. Furthermore, researchers have suggested that the novel formulations, such as baicalin preparations with magnesium or L-arginine, may improve the solubility and therapeutic efficacy of these compounds against colitis.

## 3. Flavonols with Activity Against Ulcerative Colitis

One study has reported that oral pre-treatment with kaempferol (100 or 50 mg/kg) daily for 7 days in mice with DSS-induced colitis, followed by post-treatment with the flavonoid for another 7 days, improved body weight and colon length. In contrast, colitis did not improve in Bcrp−/− -Mrp2−/− mice treated with kaempferol. Furthermore, the concentration of 100 mg/kg showed the best results in terms of the evaluated parameters. Therefore, the authors suggested that the effect of this flavonoid on colitis is highly influenced by the presence and function of the efflux transport proteins BCRP and MRP2, which play crucial roles in the pharmacokinetics and tissue distribution of kaempferol, especially in the intestine. They also anticipated that the findings of their research will help to elucidate the relevant mechanisms of action of kaempferol [78] (Table 5). Similarly, another study focused on whether pre- and post-treatment (14 or 21 days, respectively) with a kaempferol-containing diet (0.3%) ameliorated the inflammatory responses in DSS-induced ulcerative colitis mice. Under both kaempferol treatments, IL-1β, IL-6, TNF-α, iNOS, and COX-2 mRNA expression were down-regulated, and LTB4, PGE_2,_ and NO plasma levels and MPO activity were decreased in the colon. In addition, kaempferol pre-treatment up-regulated the colonic expression of TFF3 (a marker of goblet cell function) mRNA. These results indicate that kaempferol is an efficient anti-inflammatory bioactive compound that protects the colon mucosa in this model of colitis, and that pre-treatment has greater effectiveness in inhibiting some colitis-related parameters [79]. Similarly, another study reported that oral pre-treatment with kaempferol (50 mg/kg) daily for 7 days followed by post-treatment with the flavonoid for another 7 days improved the body weight and colon length; reduced the DAI; increased the mRNA expression of IL-10, claudin-1, occludin, and ZO-1; decreased serum levels of TNF-α, IL-6, IL-1β, and LPS; and suppressed mRNA expression of iNOS, MCP-1, COX-2, TNF-α, IL-6, IL-1β, NLRP3, p-NF-κB-P65, TLR4, and MyD88 in the colon of mice with DSS-induced colitis, suggesting inhibition of the LPS–TLR4–NF-κB inflammatory pathway. Therefore, the authors suggested that consuming this flavonoid could be beneficial for the prevention and treatment of colitis [80].

Qu et al. demonstrated that the oral administration of myricetin (80 mg/kg, for 7 days) increased TGF-β and IL-10 levels in DSS-induced ulcerative colitis mice. It also exerted an influence on the proportion of Treg cells, thus improving the severity of inflammation in colitis. These results suggest that this compound has protective properties against colitis, and may be considered as a candidate for treating this disease [81]. In 2025, Xu et al. reported that the daily oral administration of myricetin for 10 days at a concentration of 80 mg/kg in mice with DSS-induced colitis improved their colon length; reduced the DAI score, IL-6 and IL-17 levels, expression of the transcription factor RORγt (for Th17 cells), and the proportion of Th17 cells; and increased colonic IL-10 and IL-22 levels, expression of the transcription factor Foxp3 (for Treg cells), and the proportion of Treg cells. Furthermore, it positively modulated the AhR signaling pathway, and it was demonstrated that myricetin binds to AhR and promotes its activation. The authors indicated that this flavonoid could represent a potential novel drug for the treatment of colitis, opening new possibilities for the proposal and development of additional therapies based on natural products [82]. In another investigation, it was observed that treatment with myricetin (50, 100, or 200 mg/kg orally for 10 days) improved body weight loss in a dose-dependent manner and decreased histology scores in DSS-induced ulcerative colitis mice. In addition, this compound reduced MDA, NO, and MPO production and IL-6 and IL-1β levels, while increasing GSH and SOD activities. These results suggest that myricetin exerts effects on colitis, which may be due to its antioxidant and anti-inflammatory activities [83].

Similarly, it has been reported that administration of myricitrin (1, 3, or 10 mg/kg orally for 7 days) in DSS-induced ulcerative colitis mice results in an anti-inflammatory effect, which is mediated by the suppression of Akt/PI3K-dependent phosphorylation. Consequently, NF-κB, ERK1/2, MAPK p38, and c-JNK phosphorylation; TNF-α and IL-6 mRNA expression; and CXCL1/KC and IL-6 protein levels were decreased in the colon. These results suggest that myricitrin has anti-inflammatory activity in this model of colitis, which could be associated with its capacity to prevent the activation of NF-κB, MAPK, and PI3K-dependent Akt [84].

Xuan et al. observed that pre-treatment with galangin (15 mg/kg, orally for 7 days) reduced pathological changes in the colon, colon length shortening, and the DAI in DSS-induced ulcerative colitis mice. In addition, this compound reduced MPO activity and IL-6, IL-1β, and TNF-α levels; increased LC3B, ATG12, ATG7, and ATG5 protein expression; and stimulated the formation of autophagosomes in the colon, leading to the conclusion that galangin could be used to treat and/or prevent colitis [85]. Gerges et al. reported that galangin treatment (40 mg/kg via oral gavage for 20 days) improved tissue damage and histopathological alterations, down-regulated TLR4 expression, inhibited NF-κB (p65) activation, decreased TNF-α and IL-6 levels, and increased tissue GSH levels in DSS-induced ulcerative colitis mice, indicating that this compound as a potential candidate for the treatment of colitis [86]. In another study, it was observed that treatment with galangin (20 or 40 mg/kg orally for 12 days) decreased the severity of disease and attenuated microscopic and macroscopic clinical signs in DSS-induced ulcerative colitis mice. Furthermore, this compound decreased IL-6 and TNF-α levels in serum and colon tissue; raised IL-10 levels and SOD and reduced nitrites, TBARS, and MPO in the colon; and inhibited p-Ikk-βα and p-NF-κB, Nrf2, iNOS, and COX-2 protein expression. These results suggest that galangin has anti-inflammatory activity, and may be useful for treating colitis [87]. Another study reported that the oral administration of galangin (40 mg/kg) daily for 10 days improved body weight and colon length; reduced DAI and serum IL-1, IL-6, and TNF-α levels; and reduced the expression of cleaved caspase-1 p20, ASC, and NLRP3 (proteins related to NLRP3 inflammasome activation), as well as MPO activity, in the colon of mice with DSS-induced colitis. Furthermore, they demonstrated that this flavonoid binds to HSP90, thus decreasing its oligomerization and stability, as well as decreasing p-AKT levels. These results suggest that galangin inhibits HSP90, decreasing NLRP3 inflammasome activation mediated by fatty acid synthesis, thereby alleviating colitis in these animals and indicating its potential for use in the treatment of this disease [88].

Another study reported that the oral administration of a silk-sericin-stabilized fisetin preparation (200, 100, or 40 mg/kg) daily for 8 days improved body weight and colon length, increased SOD levels, and reduced DAI, IL-1β, and TNF-α levels in mice induced with colitis via DSS, as well as reducing activation of the cGAS/STING signaling pathway (molecules involved in inflammation). The 100 mg/kg concentration of the flavonoid showed the best results for all parameters evaluated. These results suggest that the silk-sericin-stabilized fisetin preparation could be a promising candidate for the development of natural, effective, and safe treatments for colitis [89]. Other researchers have observed that fisetin administration (5 or 10 mg/kg orally via gavage for 8 days) decreased the severity of disease and attenuated its microscopic and macroscopic signs in mice with DSS-induced ulcerative colitis. This compound reduced MPO activity; IL-6, IL-1β, and TNF-α production; and iNOS and COX-2 expression in colonic tissues. Additionally, it inhibited the activation of NF-κB (p65) by suppressing IκBα phosphorylation and NF-κB (p65)–DNA binding activity and mitigated Akt and p38 phosphorylation in the colon, while also increasing GSH and reducing MDA levels. These results suggest that fisetin’s anti-inflammatory effect is mediated by the suppression of NF-κB, p38, and Akt signaling in colonic tissues, and it could be considered as an alternative therapeutic agent for ulcerative colitis [90].

One study reported that the oral administration of zinc–rutin particles (40 mg/kg) daily for 11 days improved body weight, colon length, and the DAI; reduced the levels of TNF-α, IL-1β, and IL-6, and MDA activity; and increased CAT and SOD activities in mice with DSS-induced ulcerative colitis. Therefore, the authors concluded that the formulation of different flavonoid-loaded particles represents a potential strategy for developing safe and efficient treatments for colitis [91]. In 2024, Zhao et al. [92] reported that the oral administration of rutin (100, 50, or 25 mg/kg) daily for 7 days improved body weight, colon length, and the DAI; decreased levels of IL-6, IL-18, TNF-α, and IL-1β in the colon and serum; and decreased NLRP3 mRNA expression and protein levels of cleaved caspase-1, cleaved IL-1β, and NLRP3 in the colonic tissue of mice with DSS-induced ulcerative colitis. Therefore, the authors suggested that this flavonoid has a therapeutic effect in this animal model of colitis, mediated—at least in part—by the inhibition of inflammation and the NLRP3 inflammasome. These findings provide a basis for exploring the potential of rutin as a treatment for this disease [92]. Sharma et al. demonstrated that rutin treatment (25 or 50 mg/kg orally for 48 days) attenuated body weight loss, colon length shortening, the DAI, and goblet cell loss; reduced ICAM-1, HO-1, and iNOS mRNA levels in the colon; and up-regulated IL-6, TNF-α, and IL-1β expression in DSS-induced ulcerative colitis mice. The TNF-α/IL-10 ratio was also modulated, affecting the secretory levels of the ratios of IL-5/IL-10, IL-6/IL-10, IL-1β/IL-10, TNF-α/IL-10, and IFN-γ/IL-10, thereby regulating these cytokines to prevent inflammation and damage in the colon. In addition, rutin regulated the MAPKs/NF-κβ and p38/MK2 signaling pathways and reduced activation of the PI3K/Akt/GSK3β signaling pathway, suggesting that it could attenuate colitis through regulation of these pathways. Moreover, this compound increased B-cell lymphoma 2 (Bcl-2) and proliferating cell nuclear antigen (PCNA) expression; reduced Bcl-2-associated X (BAX) protein in intestinal epithelial cells, suggesting an effect on the preservation of homeostasis in these cells; and increased occludin, ZO-1, MUC3, and MUC2 mRNA expression in the colon, thus recovering epithelial integrity. Additionally, this compound increased the number of Treg cells and decreased CD4^+^ and CD8^+^ T cells in the spleen, thus contributing to the modulation of these cells to inhibit the severity of inflammation. This suggests that rutin can modulate the p38/MK2 and PI3K/Akt/GSK3β/NF-κB pathways in this model of colitis, which could represent a new avenue for the therapeutic treatment of this disease [93]. In 2024, Sherif et al. reported that the oral administration of rutin (100 mg/kg) daily for 10 days improved the colon weight/colon length ratio, decreased serum levels of lactate dehydrogenase (LDH) and CRP (markers of tissue damage and systemic inflammation), increased the total antioxidant status (TAS), and reduced the total oxidative status (TOS) in the colon of rats with acetic-acid-induced colitis, where TOS and TAS are indicators of oxidative stress. In addition, it decreased both the mRNA expression and protein levels of MYD88, TLR4, and HMGB1 and reduced NF-kB expression in the colon, suggesting inhibition of the HMGB1/TLR4/MYD88/NF-kB signaling pathway. These results suggest that this flavonoid could be considered as a candidate for the treatment of colitis [94].

Regarding quercetin, research on the effect of this flavonoid in the context of colitis has also increased significantly over the past 5 years. Various studies have demonstrated that the administration of quercetin in TNBS-induced [95] or acetic-acid-induced [96] ulcerative colitis rats (50 or 100 mg/kg treatment orally, for 10 days or pre-treatment for 3 days, respectively) raised the body weight, reduced rectal bleeding, and improved the length and weight of the colon, while also reducing MPO activity and raising GSH levels in the colon. These results suggest that quercetin mitigates colonic damage, probably through its antioxidant activity [95,96] (Table 6). In one study, it was demonstrated that treatment with quercetin-loaded microcapsules (1, 10, or 100 mg/kg orally) decreased macroscopic damage, histological alterations, and IL-33 and IL-1β production in the colon of mice with acetic-acid-induced ulcerative colitis. The treatment also mitigated decreases in antioxidant capacity and IL-10 in the colon. These results indicate that quercetin-loaded microcapsules have enhanced antioxidant and anti-inflammatory activities, and can be considered as a potential bioactive compound for the treatment of colitis [97].

Zhang et al. conducted a study using a DSS-induced murine model of ulcerative colitis. Once the disease was established, the animals were sacrificed on day 13, at which point intestinal samples were collected. Treatment with quercetin (100 mg/kg) demonstrated beneficial effects by reducing inflammation and cellular infiltration, as well as decreasing the expression of inflammatory factors such as IL-1β, TNF-α, and IL-6. Based on these findings, quercetin was proposed as a potential therapeutic option for the treatment of UC [98].

In 2024, a study was conducted using a mouse model in which DSS was administered for seven days. The treatment consisted of quercetin (30 mg/kg), and the mice were evaluated according to the DAI, which measures weight loss, stool consistency, and the presence of occult blood in feces. The levels of urea, nitrogen, creatinine, alanine aminotransferase (ALT), and aspartate aminotransferase (AST) were also analyzed, along with the expression of antibodies such as α-tubulin, cGAS, STING, and iNOS. The study’s results indicated that quercetin reduced intestinal inflammation by inhibiting the cGAS–STING pathway and modulating anti-inflammatory factors such as CXCL10, TNF-α, CCL17, and IL-10. Based on these findings, it was concluded that quercetin contributes to restoration of the intestinal barrier, leading to symptom relief [99].

Similarly, also in 2024, Jiang et al. administered DSS to induce UC in a mouse model. Quercetin was used as a treatment (10 mg/kg, daily intragastric administration for 11 days), resulting in reductions in the expression levels of inflammation-related proteins including CXCL8, CXCR1, CXCR2, IL-17A, and NF-κB. These observations provide support for quercetin’s protective role against UC [100]. In the same year, a research team led by Lv et al. also used DSS to induce UC in a mouse model. They evaluated the levels of IL-1β, TNF-α, IL-6, and IL-10, along with MDA, CAT, GSH, and SOD, as indicators of the severity of the disease. The study results indicated that quercetin (50 mg/kg, daily via intragastric administration for 14 days) reduced IL-1β, TNF-α, IL-6, and MDA while increasing IL-10 and GSH levels and CAT and SOD activities, thus alleviating tissue damage and protecting the intestinal barrier by reducing both the inflammation and oxidative stress caused by DSS. Furthermore, quercetin was observed to inhibit macrophage polarization, minimizing associated damage [101].

In another study conducted in 2024 by Zhao et al. [102], mice were evaluated for a period of 6 to 8 weeks. DSS was used as the induction agent, while the treatment experiment compared quercetin (45 mg/kg) with mesalazine (100 mg/kg), an anti-inflammatory drug known for its effectiveness against UC. The treatment was designed in the form of a hydrogel, in order to prolong the drug’s shelf life and protect ulcerated tissues. The mice were evaluated according to the DAI, and histological samples were analyzed to observe cellular infiltration and structural damage in the tissues. It was observed that, under treatment with the hydrogel, the architecture of the intact colonic tissue closely resembled that of the control group, with minimal distortion of the crypts and less inflammatory cell infiltration. In addition, the levels of proteins such as claudin-1, occludin-1, and ZO-1 were determined to evaluate the integrity of the intestinal mucosa, with increases in these proteins observed under the proposed treatment. Proinflammatory cytokines such as TNF-α and IL-6 were also analyzed, with were observed to decrease after treatment with the hydrogel, even surpassing the decrease under the mesalazine-based treatment. The study results indicated that the hydrogel can effectively contribute to disease reduction, restoration of the intestinal mucosa, prevention of colonic cell apoptosis, and mitigation of the oxidative stress caused by UC.

In a study conducted in 2025, DSS-induced mice were treated for a period of 7 to 8 weeks. The treatment consisted of mesalazine or one of two quercetin concentrations: high (50 mg/kg) or low (20 mg/kg). The mice were evaluated according to the DAI and histopathological analysis. The results demonstrated that high-dose quercetin significantly ameliorated weight loss and colon length and reduced the DAI, similarly to the mesalazine-treated group. Tissue damage was observed in the UC mice, whereas those in the quercetin and mesalazine groups showed less damage. Furthermore, the study revealed an increased number of goblet cells and higher mucin levels in the treatment groups compared with the UC group. In this way, it was confirmed that quercetin effectively ameliorated fibrosis-associated structures compared with the UC group. Moreover, tight junction protein expression was evaluated, revealing significant increases in occludin, claudin-1, and ZO-1 in the high-dose quercetin group, suggesting that quercetin is effective in repairing the intestinal barrier and maintaining colon function after DSS-based disease induction [103].

Dong et al. reported that administering a quercetin-containing diet (500–1500 ppm) for 6 days decreased body weight loss, colon length shortening, and the histopathological score in DSS-induced ulcerative colitis mice. Furthermore, this compound attenuated intestinal permeability and increased CAT and SOD activities and GSH levels [104]. Riemschneider et al. comparatively investigated the effects of quercetin in DSS-induced ulcerative colitis mice and Ahr−/− mice. The quercetin treatment (50 mg/kg via oral gavage for 10 days) decreased the histopathological score; ameliorated the clinical symptoms of colitis; induced the expression of the tight junction protein claudin-1, partially restoring the loss of epithelial integrity; and raised the number of Treg cells while decreasing the number of Th17 cells. On the other hand, there was no improvement of colitis symptoms in Ahr−/− mice treated with quercetin, indicating the key roles of AhR ligation and signaling. This outcome demonstrates that further studies on quercetin are still necessary to determine its effectiveness, dose–response effects, and probable adverse effects; however, it still shows potential as a candidate for treating colitis in humans [105].

Diez-Echave et al. [106] evaluated the anti-inflammatory effects of fibroin nanoparticles loaded with quercetin (QSFNs) in a DSS-induced colitis mouse model. Considering the well-known low bioavailability of quercetin, which conditions its therapeutic applicability, nanotechnology-based systems have been suggested as a strategy to improve the biological effects of quercetin against gastrointestinal pathologies. The fibroin nanoparticles loaded with quercetin (5 mg/kg, orally daily for 7 days) were found to reduce DAI values on the 6th day of treatment, with respect to the control group. However, in the early days of the treatment, there were no significant differences among the experimental groups. Although the authors of this work focused on QSFNs to enhance the macroscopic effects of quercetin in comparison to quercetin or the fibroin nanoparticles alone, a statistical analysis was not conducted. Regardless, a histological analysis revealed that the QSFNs preserved the normal crypt microarchitecture in the colons of mice, increased the mucin content, and decreased inflammatory infiltration in the mice in comparison to the control group and the quercetin- and fibroin nanoparticle-only groups, which was associated with the intestinal anti-inflammatory effects of the QSFNs. As a result of the lower inflammatory infiltration in the colons of mice treated with the flavonoid-loaded nanoparticle system, lower colonic expression of MCP-1 (a chemotactic protein) and ICAM-1 (an adhesion molecule that participates in lymphocyte migration on inflamed tissues) was observed.

The fibroin nanoparticles alone displayed a similar inhibitory effect on TNF-α and IL-1β and increased the expression of IL-6 cytokines more than the QSFNs, indicating that the nanoparticles alone contribute to the anti-inflammatory effects of the flavonoid-loaded nanoparticle system; this is in contrast to the activity observed with quercetin alone, which had no effects on these biomarkers. Additionally, the authors reported that treatment with quercetin alone only significantly reduced the NLRP3 inflammasome, which is a regulator of intestinal homeostasis [107]. Furthermore, when iNOS was measured in the colitis model mice, QSFNs were the only treatment that reduced its expression. Therefore, the use of this type of flavonoid-loaded nanocarrier system may play a crucial role in enhancing the beneficial effects of flavonoids against these types of pathologies [106].

Nanoparticles loaded with flavonols have also been utilized by other authors; for example, chitosan quercetin-loaded nanoparticles (QT-NPs) have been shown to enhance the anti-colitic properties of this compound [108,109]. Oral administration of these nanoparticles in DSS-induced colitis rats restored the body and spleen weight lost after DSS treatment; moreover, a macroscopic analysis revealed that the QT-NPs treatment (20 mg/kg, orally daily for 14 days) maintained a similar colon length to that observed in the non-DSS-induced control rats. Consequently, the DAI parameter was reduced in a dose-dependent manner with the quercetin nanoparticles, when compared with the untreated DSS-induced colitis rats [108]. Similarly, in 2024, Wang et al. [109] found that QT-NPs (200 mg/kg, orally daily for 21 days) decreased the most commonly observed alterations in mice with colitis, such as weight loss, fecal hemorrhage, diarrhea, splenomegaly, inflammatory cell infiltration, shortened colon length, and abnormal mucus secretion by goblet cells.

Regarding the implications of treatment with QT-NPs in the colitis murine model used in this work, the authors found that this quercetin nano-formulation reduced the level of fecal calprotectin [108], a biomarker of inflammatory bowel disease that increases during active inflammatory periods and, therefore, indicates mucosal intestinal neutrophil migration, as a response to intestinal injury [110]. In this sense, the authors attributed this effect to particle size reduction to a nanometric scale, which can enhance the bioavailability and dispersion of active principles such as quercetin. Additionally, the administration of QT-NPs in the DSS-induced colitis model restored the levels of red blood cells and hemoglobin, and kidney and liver tissue-protective effects were observed as a consequence of the antioxidant activity of the quercetin chitosan nano-formulation. Furthermore, the QT-NP treatment reduced ROS and H_2_O_2_ levels in colitic rats in a dose-dependent manner, indicating that it reduces lipid peroxidation and free radical content and stimulates the antioxidant defense system in colitic rats [108]. This is related to the capacity of the QT-NPs to up-regulate the expression of HO-1 and Nrf2, key components of the nuclear factor erythroid 2-related factor 2-mediated antioxidant system, which protects cells against oxidative stress [111] in colitic rats.

In addition, QT-NPs have been shown to reduce MPO activity in DSS-induced colitis rats [108] and mice [109]. MPO activity is a marker for leukocyte sequestration in colonic tissue and, therefore, is related to disease progression. In this sense, QT-NPs can lower the levels of the proinflammatory cytokines IL-6, IL-1β, IFN-γ, and TNF-α and, in contrast, can increase the level of the anti-inflammatory cytokine IL-10 in colitis models in rats and mice [108,109], as reflected by the amelioration of colonic epithelial damage.

Kahter et al. [108] have proposed that the increased level of IL-10 observed in DSS-induced colitis rats treated with QT-NPs could be a compensatory mechanism for an inflammatory response in the colonic tissue, which possibly plays a key role in limiting the inflammatory process through a reduction in MHC class II antigen production; this is reflected by the enhancement of inflammatory processes in the context of colon injury. In support of this idea, treatment of colitic rats with QT-NPs also down-regulated CD4 and CD8 gene expression, which decreases excessive inflammatory reactions to suppress the maturation of T lymphocytes [108]. Furthermore, Wang et al. [109] and Wang et al. [112] reported that the administration of quercetin alone or chitosan and quercetin-loaded nanoparticles increased the expression of the tight junction proteins ZO-1, claudin-1, and occludin, which have been implicated in the integrity and selectivity of the intestinal epithelium. Moreover, Wang et al. [112] reported that quercetin alone restored tight junction integrity via an AhR-dependent mechanism. AhR is a regulator of intestinal barrier homeostasis and, possibly, tight junction protein expression [113,114]; therefore, its activation may enhance the expression of the ZO-1 and occludin, while also inhibiting the expression and phosphorylation of myosin light chain kinase, as evidenced by the restoration of tight junctions of the intestinal epithelial barrier [115,116]. This is reflected in the enhanced therapeutic effect of QT-NPs on intestinal barrier integrity [109], with respect to quercetin alone [112].

The relevance of considering nanoparticle systems loaded with quercetin is demonstrated through a work in which a 0.45% quercetin diet with pectin at 6% as the fiber source (emulating the characteristics of the fiber content in stone fruits) was evaluated in a DSS-induced colitis rat model. The authors reported an increase in the concentration of short-chain fatty acids when they stimulated the formation of colitis in rats with DSS; this change was attributed to an alteration in the intestinal microbiota of the rats, in agreement with the results reported by Wang et al. [109] in a mouse model of DSS-induced colitis. Although treatment with the chitosan nanoparticles loaded with quercetin resulted in a significant reduction in Protobacteria in comparison with the colitic mice treated with quercetin alone, the abundance of Lactobacillus did not revert to normal levels.

Although the quercetin diet did not decrease intestinal injury and inflammation scores in the DSS-induced colitis model rats, it maintained similar butyrate levels in colitic rats with respect to the non-colitic rats. This result is relevant, as increased levels of butyrate can become toxic with the progression of ulcerative colitis; therefore, quercetin may diminish the damage caused by elevated intestinal tissue levels of butyrate in this acute model of colitis. Furthermore, a quercetin-enriched diet has been shown to be useful in terms of enhancing the levels of injury repair molecules and suppressing inflammation-related pathways [117]. This idea is supported by other research In which a quercetin diet strengthened the expression of FGF-2 in colitic rats, which helps to rebuild intestinal tissue from the basolateral membrane; as DSS induces a decrease in TFF-3, is an apical wound-healing molecule expressed by goblet cells [118], the repair of intestinal tissue is expected to be more critical in deeper tissues than in the apical intestinal tissue [117].

Quercetin mitigates the reduced expression of TNFR-1, which can induce cell death in intestinal tissue that contributes to UC-related damage caused by DSS treatment in rats. Furthermore, this flavonoid reduces the expression of TNFR-2, which plays a role in the proliferation and survival of cells that, in principle, could be beneficial but, in UC, can lead to tumorigenic processes in the colon [119]. Additionally, quercetin can regulate NF-kB, as evidenced by its capacity to maintain Tollip (Toll-interacting protein) and IkB expression and reduce the increase in the level of IL-1 (a proinflammatory cytokine) in a DSS-induced colitis rat model. Therefore, quercetin may regulate the inflammatory response in the DSS-induced colitis model through the TLR and NF-κB pathways [117].

Another strategy to improve the biological effects of quercetin is the oral administration of this flavonol; for example, it has been administered in the form of a water-soluble inclusion complex with hydroxypropyl-b-cyclodextrin in a DSS-induced colitis mouse model, in which a dose of 0.5 mg/kg of this complex reduced the DAI in untreated mice. Nevertheless, this quercetin formulation did not have any effect on weight reduction in the mice with colitis; in contrast, the reduction in bowel length was less severe in mice treated with quercetin, when compared to untreated colitic mice [120]. These results are explained by the ability of quercetin to increase the histological level of CD11b, a subunit of the integrin Mac-1 which is present on immune cells like macrophages, dendritic cells, and B cells of the peritonea; notably, this subunit is involved in the enhancement of colitis through IL-10 production [121].

**Table 6:** Effects of quercetin in different models of colitis.

Flavonol	Model	Properties	Ref.
Quercetin	TNBS/rat	Raises the body weight and GSH levels; reduces rectal bleeding and MPO activity; improves the length and weight of the colon.	[95]
	Acetic acid/rats		[96]
	Acetic acid/mice	Decreases IL-33 and IL-1β production; prevents decreases in antioxidant capacity and IL-10.	[97]
	DSS/mice	Decreases the expression of inflammatory factors such as IL-1β, TNF-α, and IL-6.	[98]
		Reduces intestinal inflammation by inhibiting the CGAS–STING pathway; modulates anti-inflammatory factors such as CXCL10, TNF-α, CCL17, and IL-10.	[99]
		Reduces expression levels of the inflammation-related proteins CXCL8, CXCR1, CXCR2, IL-17A, and NF-κB.	[100]
		Reduces IL-1β, TNF-α, IL-6, and MDA; increases IL-10 and GSH levels and CAT and SOD activities; inhibits macrophage polarization.	[101]
		Contributes to disease reduction; increases claudin-1, occludin-1, and ZO-1 expression; reduces TNF-α and IL-6 levels.	[102]
		Ameliorates weight loss, colon length, and fibrosis-associated structures; reduces the DAI; increases the expression of occludin, claudin-1, and ZO-1, number of goblet cells, and levels of mucin.	[103]
		Decreases body weight loss and colon length shortening; attenuates intestinal permeability; raises CAT and SOD activities and the GSH level.	[104]
		Decreases histopathological score and the number of Th17 cells; induces expression of claudin-1; increases the number of Treg cells; no improvement in colitis symptoms in Ahr−/− mice.	[105]
		QSFN treatment reduces DAI; preserves the normal crypt microarchitecture in colons of mice; maintains lower colonic expression of MCP-1; inhibits TNF-α and IL-1β expression; increases IL-6 expression; reduces iNOS expression.	[106]
		QT-NPs decrease MPO activity and IL-6, IL-1β, IFN-γ, and TNF-α levels; increases IL-10 level and ZO-1, claudin-1, and occludin expression.	[109]
		Increases expression of ZO-1, claudin-1, and occludin; restores tight junction integrity via an AhR-dependent mechanism.	[112]
		Reduces the DAI; improves colon length.	[120]
	DSS/rats	Diet with quercetin enhances the expression of FGF-2; mitigates the reduced expression of TNFR-1; maintains the regulation of NF-κB through Tollip and IκB expression; reduces IL-1 level.	[117]
		QT-NPs restore body and spleen weight, red blood cells, and hemoglobin; maintain colon length; reduce the levels of MPO, ROS, and H_2_O_2_; up-regulate the expression of HO-1 and Nrf2; reduce the levels of IL-6, IL-1β, IFN-γ, and TNF-α; increase the IL-10 level; down-regulate CD8 and CD4 gene expression.	[108]

Quercetin is considered a significant therapeutic option for intestinal regulation and microbiota balance, and has been observed to reduce inflammation and cellular infiltration in different murine models of ulcerative colitis. Various studies have indicated that quercetin may act by inhibiting inflammatory pathways such as CGAS–STING, modulating anti-inflammatory and antioxidant factors, and affecting the expression of inflammation-related proteins such as IL-1β, TNF-α, and IL-6. Additionally, quercetin has been shown to aid in intestinal barrier restoration by promoting mucosal repair and increasing the expression of tight junction proteins (e.g., claudin-1, occludin-1, and ZO-1), thus reducing inflammation and tissue damage. In some studies, administration of quercetin in various forms (e.g., hydrogels) resulted in effects that were comparable (or even superior) to mesalazine in terms of reducing inflammation and maintaining intestinal integrity.

Furthermore, although quercetin is well-known for its anti-inflammatory properties, it has limited bioavailability. In this regard, it has been demonstrated that the loading of quercetin onto fibrin or chitosan nanoparticles enhances its efficacy in treating ulcerative colitis in murine models. QSFNs and QT-NPs have been shown to decrease intestinal inflammation by reducing inflammatory cell infiltration, proinflammatory protein expression (e.g., MCP-1 and ICAM-1), and cytokine activity (e.g., TNF-α and IL-1β). The administration of nanoparticles loaded with quercetin in colitis model animals has provided evidence for their antioxidant effects, achieved through lowering ROS and H_2_O_2_ levels, increasing the activities of enzymes such as SOD and CAT, and regulating antioxidant factors such as HO-1 and Nrf2. Regarding the immune system, quercetin has been reported to modulate immune responses by reducing the gene expression of CD4 and CD8 while increasing IL-10 levels, contributing to regulation of inflammation in the colon. Finally, although the findings published to date are promising, further studies will be necessary in order to better understand the optimal dosages, mechanisms of action, and potential effects of these therapeutic options in humans.

Collectively, the evidence summarized above indicates that the effects of flavonols in experimental models of ulcerative colitis are not restricted to the modulation of individual inflammatory or oxidative stress markers, but involve interconnected signaling pathways that contribute to the regulation of intestinal inflammation and epithelial barrier integrity. In particular, NF-κB appears to represent a central inflammatory node that can be modulated through upstream pathways such as TLR4/MyD88, PI3K/Akt, and MAPK. For example, kaempferol was reported to suppress the LPS–TLR4–MyD88–NF-κB inflammatory pathway, resulting in reduced expression of proinflammatory mediators such as TNF-α, IL-6, and IL-1β [80]. Similarly, myricitrin was shown to exert anti-inflammatory effects through inhibition of PI3K/Akt-dependent phosphorylation, accompanied by the reduced activation of NF-κB, ERK1/2, p38 MAPK, and JNK, as well as decreased expression of TNF-α and IL-6 [84]. Rutin also modulated the MAPK/NF-κB, p38/MK2, and PI3K/Akt/GSK3β signaling pathways, together with increasing anti-inflammatory responses and the restoration of intestinal epithelial integrity [93]. In parallel, modulation of the NLRP3 inflammasome may contribute to reductions in IL-1β production and inflammatory tissue damage, as reported for galangin, rutin, and quercetin [88,92,107]. The antioxidant response mediated by Nrf2 may also interact functionally with inflammatory signaling pathways, thus restoring cellular redox homeostasis and reducing oxidative conditions associated with intestinal inflammation, as suggested in studies using quercetin-based formulations [108,109]. Furthermore, the effects of myricetin and quercetin have been associated with AhR signaling, particularly in relation to the Treg/Th17 balance and intestinal barrier homeostasis [82,105,112]. Thus, these pathways may converge functionally to regulate oxidative stress, inflammatory responses, immune cell activity, and epithelial integrity. However, the available evidence does not establish a single hierarchical signaling cascade for all flavonols; rather, the observed effects appear to result from compound-specific interactions with molecular targets together with indirect consequences of antioxidant and anti-inflammatory activities. Although direct molecular interactions have been demonstrated for some flavonols—such as the interaction of myricetin with AhR and galangin with HSP90 [82,88]—in many studies, the proposed mechanisms are inferred from changes in downstream signaling proteins, cytokines, and oxidative stress markers. Therefore, further mechanistic studies are required to determine the extent to which these compounds directly interact with specific molecular targets and the interconnections between redox regulation, NF-κB/MAPK signaling, NLRP3 activation, and AhR-mediated responses in the context of flavonol-mediated protection against experimental colitis.

One of the most recurring challenges and concerns regarding the use of natural products and their derivatives is their potential toxicity following consumption; despite their widespread use, they remain predominantly the subject of preclinical study and, to a lesser extent, human trials. Within the context of this review, quercetin is noteworthy, as a flavonoid which has been widely studied that is distributed throughout the plant kingdom. Data regarding this flavonoid are controversial. For instance, Cunningham et al. [122] analyzed male and female CD2F1 mice fed a quercetin-enriched diet (providing daily doses of approximately 12.5, 25, or 50 mg/kg of body weight) for 14 weeks, and their findings demonstrated that quercetin caused no toxic effects and did not significantly alter the levels of alkaline phosphatase (ALP), AST, and ALT. However, other important aspects of this flavonoid must be considered, particularly its effects on drug-metabolizing enzymes and transporters such as CYP3A1, CYP3A2, UGT1A1, SULT1A1, P-gp, BCRP, and MRP2. Liu et al. [123] reported that co-administering quercetin (25, 50, or 100 mg/kg) with cyclosporine in male Sprague Dawley rats for seven days altered the function of these enzymes, thus reducing the drug’s bioavailability. These studies demonstrate that there are still significant gaps in our understanding regarding flavonoids and their effects on pharmacokinetics and bioavailability when interacting with other drugs. Furthermore, evidence regarding these types of interactions exists for many drugs, which reinforces the need to continue studying natural products and their derivatives.

Although nanoencapsulation can enhance the stability and bioavailability of flavonoids, the nanomaterials used as vehicles could themselves exhibit toxicological or immunological effects, the magnitude of which depends on factors such as their composition, size, surface charge, and other physicochemical properties [124], making further research into nanoparticles crucial for their optimal application in humans.

While determining a safe and effective dosage for flavonoids in humans is a complex task, the potential reward is invaluable. It remains to be determined whether the consumption of propolis or other flavonoid-rich bee products could serve as a complementary option to therapies for UC.

Flavonols such as kaempferol, myricetin, myricitrin, galangin, fisetin, and rutin show promise for the treatment of colitis, due to their potent anti-inflammatory and antioxidant activities. As mentioned above, they contribute to reductions in the levels of inflammatory markers (e.g., TNF-α, IL-1β, IL-6), inhibition of proinflammatory pathways (e.g., NF-κB, MAPK, NLRP3 inflammasome), modulation of immune responses (e.g., increase in Treg cells), and strengthen the intestinal barrier by improving the expression levels of tight junction proteins and goblet cell function. Bioavailability remains a key factor, and nanoparticle formulations involving quercetin or zinc–rutin particles have demonstrated superior efficacy to either the compound or particle alone, offering improved colonic protection and significant relief from colitis symptoms.

## 4. Flavanones with Activity Against Ulcerative Colitis

In 2024, Abdollahy et al. reported that the oral administration of Eudragit^®^ S100/chitosan nanoparticles (made primarily of a chitosan matrix ionically crosslinked with the anionic copolymer Eudragit^®^ S100 (methyl methacrylate and methacrylic acid)) loaded with hesperidin (8 mg/kg) daily for 7 days improved body weight and colon length and reduced the DAI, percentage ulcer area, and ulcer index in rats with acetic-acid-induced ulcerative colitis (Table 7). Their results suggest that the flavonoid-loaded nanoparticles did not show toxicity while increasing the bioavailability of hesperidin, which could offer a promising therapeutic strategy for UC [125].

In another investigation, it was observed that naringenin administration (50 or 100 mg/kg via gavage for 7 days) raised the colonic mucus content, increased SOD and CAT activities, and reduced TBARS levels in acetic-acid-induced ulcerative colitis rats in a dose-dependent manner. Furthermore, this compound reduced IL-6, IL-1β, and TNF-α levels and NO and PGE_2_ values. These results suggest that naringenin has anti-inflammatory and antioxidant properties, conferring protective activity against colitis [126].

One study reported that naringin pre-treatment (40 or 80 mg/kg orally for 7 days) raised GSH and SOD in the colon of acetic-acid-induced ulcerative colitis rats. Furthermore, this compound reduced the ulcer area, macroscopic score, MPO activity, and colonic MDA and suppressed LDH and ALP serum levels in a dose-dependent manner. Additionally, this compound reduced DNA damage, protein carbonyl content, and xanthine oxidase and NO levels in the colon. These results suggest that naringin presents potent antioxidant, anti-apoptotic, and anti-inflammatory activities in some colon sites, as it regulates the expression and production of different oxidative mediators, thus decreasing DNA damage [127].

Further exploring the therapeutic potential of naringin, Dong et al. [128] evaluated its effects in two well-established murine models of colitis, namely, those induced by DSS and TNBS, both of which replicate key features of human inflammatory bowel disease. In both DSS- and TNBS-induced colitis models, low- (20 mg/kg) or high-dose (40 mg/kg) naringin was administered via a nasogastric tube daily for seven days, starting one day after colitis induction. DSS was administered in drinking water, while TNBS was administered via intrarectal injection. An additional group in the DSS model received the PPAR-γ inhibitor BADGE to assess the role of PPAR-γ.

The results of this study indicated that, while administration of naringin in healthy mice had no significant effects on their body weight, food intake, cytokine levels, or PPAR-γ expression (except at a very high dose of 160 mg/kg, which reduced PPAR-γ expression), it significantly improved multiple disease parameters in both colitis models. Notably, it reduced the DAI, mitigated body weight loss, and partially restored the colon length, suggesting overall amelioration of intestinal inflammation. Furthermore, naringin decreased the activity of MPO, a marker of neutrophil infiltration, and suppressed the expression of key proinflammatory mediators such as TNF-α, IL-1β, and IFN-γ. Regarding oxidative stress, naringin reduced the iNOS and p-NF-κB-p65 levels, although no specific antioxidant enzymes (e.g., SOD or CAT) were quantified in vivo. Likewise, while tight junction proteins such as ZO-1 or occludin were not directly assessed, improvements in epithelial barrier function were observed. At the molecular level, transcriptomic analysis revealed that naringin modulates several signaling pathways, including those involved in calcium signaling, neuroactive ligand–receptor interactions and, notably, the PPAR-γ pathway. The up-regulation of PPAR-γ was associated with inhibition of NF-κB activation, suggesting a key anti-inflammatory mechanism of action. These findings highlight PPAR-γ as a central molecular target of naringin in experimental colitis, and underscore it as a multifaceted natural compound with potential for the management of intestinal inflammation [128].

Hambardikar and Mandlik [129] investigated the protective effects of naringin in a TNBS-induced UC model in rats. In this in vivo experiment, three treatment groups orally received the flavonoid at doses of 20, 40, or 80 mg/kg, administered once daily for 14 consecutive days. UC was induced via a single intra-rectal administration of TNBS (100 mg/kg) delivered via a medical-grade catheter. 

The study demonstrated that this compound significantly ameliorated the severity of disease, as evidenced by improvements in clinical symptoms (including reduced rectal bleeding and diarrhea and improved stool consistency). Furthermore, treatment with this compound at doses of 20, 40, and 80 mg/kg effectively inhibited colonic erosion, necrosis, ulcer formation, and inflammatory infiltration and maintained normal body weight when compared to the control group. Furthermore, the treatment of rats with this flavonoid at doses of 40 and 80 mg/kg for 14 days significantly increased body weight and considerably reduced colon weight and the colon weight-to-length ratio compared to control rats, indicating the attenuation of inflammation and edema; however, the lower dose of naringin (20 mg/kg) did not produce significant effects on these parameters. Concerning proinflammatory cytokines (in this case, serum levels of TNF-α and IL-12), treatment with naringin at doses of 40 and 80 mg/kg resulted in significant reductions in these cytokines, indicating an anti-inflammatory effect. Additionally, naringin was found to restore antioxidant defense systems by increasing colonic levels of SOD, GSH, and CAT, while reducing markers of oxidative damage such as MDA and MPO. Furthermore, histopathological analyses revealed that naringin at 40 and 80 mg/kg mitigated colonic tissue damage, preserved goblet cell integrity, reduced epithelial necrosis and inflammatory cell infiltration, and restored the mucosal architecture. Although specific molecular pathways were not directly assessed, the observed modulation of cytokine levels and histological improvements suggest the suppression of inflammatory signaling, potentially involving the NF-κB pathway. Moreover, naringin appeared to preserve epithelial barrier function by enhancing goblet cell activity and reinforcing the mucus layer [129], indicating the maintenance of tight junction integrity [130]. The authors concluded that these findings collectively support naringin as a promising multi-target therapeutic candidate for the treatment of UC [129].

One study reported that hesperetin administration (100 mg/kg via oral gavage for 10 days) decreased CD45, TNF-α, Bax, and caspase-3 expression in colonic tissue of TNBS-induced ulcerative colitis rats, indicating that this compound has anti-apoptotic and anti-inflammatory activities and, thus, may prevent colon damage [131]. In another investigation, it was reported that the oral administration of hesperetin (100 or 50 mg/kg) daily for 7 days improved body weight; reduced the colonic macroscopic damage score; elevated the GSH content and SOD activity; decreased the NO content, IL-6 level, MPO activity, ICAM-1, TNF-α, RAGE, and NF-κB expression, and p-STAT3 and p-JAK2; and increased SOCS3 (suppressors of cytokine signaling 3) expression in the colon of rats with TNBS-induced ulcerative colitis. Furthermore, the effect of this flavonoid was dose-dependent, with the highest dose tested showing the best results. The authors concluded that hesperetin has effects on colitis in these model animals, partly through the inhibition of oxidative stress and inflammation and modulation of the JAK2/STAT3/SOCS3 signaling pathway. Therefore, this flavonoid could be considered for evaluation as a therapeutic agent for colitis [132].

In another investigation, administration of naringenin or hesperetin (40 mg/kg) orally for 7 days before and 3 days after TNBS-induced ulcerative colitis in mice mitigated colon shortening and body weight loss, reduced the DAI, and up-regulated the expression of ZO-1, occludin, and claudin-2. These results suggest that both compounds have modulatory activity in the context of colitis, which is mediated through the protection of colon mucosal integrity, and thus could be considered as candidates for the treatment of this disease [133].

**Table 7:** Effect of various flavanones in different models of colitis.

Flavanone	Model	Properties	Ref.
Hesperidin	Acetic acid/rats	Improves body weight and colon length; reduces the DAI, percentage ulcer area, and ulcer index.	[125]
Naringenin		Increases the colonic mucus content and SOD and CAT activities; reduces TBARS, IL-6, IL-1β, and TNF-α levels and NO and PGE_2_ values.	[126]
Naringin		Increases GSH and SOD; reduces ulcer area, macroscopic score, MPO activity, and MDA; suppresses LDH and ALP serum levels; reduces DNA damage, protein carbonyl content, and xanthine oxidase and NO levels.	[127]
	DSS/mice TNBS/mice	Mitigates body weight loss; improves colon length; decreases the DAI, TNF-α, IL-1β, IFN-γ, and iNOS levels, and MPO activity; modulates the PPAR-γ pathway and inhibits NF-κB activation.	[128]
	TNBS/rats	Reduces TNF-α and IL-12 levels; increases SOD, GSH, and CAT levels; decreases MDA and MPO; preserves mucosal integrity by maintaining goblet cell function and potentially modulating the NF-κB signaling pathway.	[129]
Hesperetin		Decreases CD45, TNF-α, Bax, and caspase-3 expression.	[131]
		Improves body weight; reduces colonic macroscopic damage score; increases GSH content and SOD activity; decreases NO content, IL-6 levels, MPO activity, ICAM-1, TNF-α, RAGE, and NF-κB expression, and p-STAT3 and p-JAK2; increases SOCS3 expression in the colon, suggesting modulation of the JAK2/STAT3/SOCS3 signaling pathway.	[132]
Naringenin and hesperetin	TNBS/mice	Mitigate colon shortness and body weight loss; reduce DAI; up-regulate ZO-1, occludin, and claudin-2 expression.	[133]

In one study, it has been reported that pre-treatment with pinocembrin (5 or 10 mg/kg, orally for 7 days) improved clinical symptoms, the loss of intestinal goblet cells, and colonic pathological damage; inhibited TNF-α, IL-6, and IL-1β gene expression; and raised occludin and ZO-1 protein gene expression in the colon of DSS-induced ulcerative colitis rats. This suggests that pinocembrin has a wide range of protective activities in this model of colitis, which could be leveraged to prevent and/or treat this disease [134] (Table 8). Yue et al. have reported that pinocembrin treatment (50 or 100 mg/kg via oral gavage for 9 days) alleviated the severity of DSS-induced ulcerative colitis in mice by decreasing body weight loss, blood in stool, diarrhea, and colon shortening. Moreover, this compound reduced TLR4 protein levels, p65 phosphorylation, and Myd88, TLR4, iNOS, TNF-α, and COX-2 mRNA expression levels in the inflamed colon. These results suggest that pinocembrin may mitigate colitis—at least in part—by inhibiting the abnormal activation of the TLR4/NF-κB signaling pathway [135].

Similarly, it has been reported that treatment with eriocitrin (30 mg/kg via oral gavage for 7 days) decreased colon histopathological damage, colon shortening, and body weight loss, as well as the secretion of TNF-α, IL-1β, IL-6, and MPO and the accumulation of infiltrated inflammatory cells in the inflamed area, in DSS-induced ulcerative colitis mice. As such, this compound shows potential for the mitigation of colitis [136].

Various studies have confirmed the therapeutic potential of naringenin in UC; for example, in 2025, Li et al. [137] evaluated its protective role in a murine model of DSS-induced UC. Oral administration of naringenin at doses of 25, 50, and 100 mg/kg over seven consecutive days significantly alleviated body weight loss, reduced the DAI, mitigated colon shortening, and decreased spleen enlargement compared to the control group. Histological analysis demonstrated preservation of the crypt architecture, improved mucosal integrity, and reduced infiltration of inflammatory cells, accompanied by a dose-dependent decrease in the tissue damage index. These beneficial effects correlated with the up-regulation of antioxidant-related proteins such as Nrf2, HO-1, and NQO1 and the simultaneous down-regulation of proinflammatory markers including NF-κB, TNF-α, and IL-1β. Notably, the co-administration of ML385—an Nrf2 inhibitor—partially reversed these effects, confirming the central role of this transcription factor in mediating the compound’s activity. Moreover, network pharmacology and bioinformatics analyses revealed that the regulated genes are enriched in biological pathways associated with oxidative stress, immune modulation, and inflammation. Complementary molecular docking, dynamic simulation, and surface plasmon resonance (SPR) analyses demonstrated strong and specific binding affinity to Nrf2, reinforcing its mechanistic relevance. Together, these findings provide deeper insight into the multitarget molecular actions of this flavonoid and highlight its capacity to activate the Nrf2 signaling pathway while suppressing NF-κB-mediated inflammation. This dual modulatory effect underscores naringenin’s therapeutic potential and supports its use as part of a complementary strategy for the management of UC [137].

In another study, it was observed that the administration of a naringenin-containing diet (0.3%) for 11 days in DSS-induced ulcerative colitis mice decreased body weight loss and inhibited TNF-α mRNA expression. Furthermore, this compound increased junctional adhesion molecule-A and claudin-3, as well as CD206 and IL-10 mRNA expression in the colon. In addition, naringenin decreased TNF-α-positive epithelial cells and stimulated the polarization of M2-type macrophages in the colon, indicating that this compound can promote recovery from colonic damage, likely through a reduction in the TNF-α level and the polarization of M2-type macrophages [138]. Similarly, it has been reported that treatment with a diet containing naringenin (0.3%) for 9 days decreased colon shortening, the DAI, and the gene expression of IL-17A, IL-6, and MIP-2 in DSS-induced ulcerative colitis mice. Additionally, the naringenin-containing diet was found to increase the expression of tight junction barrier proteins such as claudin-3, junctional adhesion molecule-A, and occludin. These results suggest that this compound ameliorated colitis—at least in part—through its anti-inflammatory activity and protection of the tight junction barrier [139]. In another study, it was demonstrated that pre-treatment with naringenin (50 mg/kg orally for 10 days) decreased the effects of DSS-induced ulcerative colitis in mice, as evidenced by down-regulation of MCP-1, ICAM-1, iNOS, COX-2, IL-6, and TNF-α mRNA in the colonic mucosa. Additionally, the decreases in IL-6 and TNF-α were linked to reductions in mucosal TLR4 mRNA and protein. Naringenin also reduced p-NF-κB (p65) protein levels, which was linked to a reduction in p-IκBα protein. These results suggest that naringenin exerts a suppressive effect on TLR4/NF-κB pathway activation in the context of the considered model of colitis [140].

Further research on the naringin has been reported in a recent study by Fan et al. [141], published in 2024. In this study, novel, orally bioavailable, and colon-targeted nanoparticles were successfully designed for the precise delivery of naringin to alleviate UC in mice. In this in vivo investigation, the authors evaluated the therapeutic efficacy of naringin-loaded nanoparticles (MC@Nar-NPs) in a DSS-induced murine model of UC. These nanoparticles consisted of a PLGA–PEG copolymer core encapsulating naringin, which were surface-modified with chitosan and mannose to enhance mucosal adhesion and macrophage targeting [142]. In the experimental design, UC was induced by administering DSS for five days. From day 1 to day 7, mice received daily oral gavage of MC@Nar-NPs at a dose equivalent to 10 mg/kg of naringin. After DSS withdrawal and a 72 h recovery period, animals were euthanized on day 8 for analysis.

The treatment led to significant improvements in clinical and histological parameters. Mice treated with MC@Nar-NPs exhibited reduced DAI scores, mitigated body weight loss, and preserved colon length. Histological evaluation showed alleviated colonic damage, reduced epithelial disruption, and restoration of goblet cells. At the molecular level, MC@Nar-NPs significantly down-regulated proinflammatory cytokines (IL-6, IL-1β, and TNF-α) and increased the activities of antioxidant enzymes (including SOD, CAT, and GSH), effectively lowering ROS levels in colonic tissues. Treatment also enhanced intestinal barrier function by up-regulating the expression of the tight junction protein ZO-1 and decreasing epithelial apoptosis [143,144]. The anti-inflammatory effect observed after MC@Nar-NP treatment resulted from the inhibition of NLRP3 inflammasome activation and possible modulation of the NF-κB signaling pathway—both of which are central regulators of intestinal immune and inflammatory responses [145]. Importantly, no signs of systemic toxicity or liver damage were observed, supporting the biosafety of the nanoparticle system. In summary, the presented findings support the use of MC@Nar-NPs as a valuable approach in the development of targeted and multi-faceted therapies for the management of UC [141].

Cao et al. [146] investigated the protective effects of naringin against DSS-induced UC in mice and the underlying mechanisms. The treatment included different doses of naringin (25, 50, and 100 mg/kg), administered orally for seven days. Naringin significantly attenuated the severity of disease, as evidenced by dose-dependent reductions in the DAI. Moreover, it effectively mitigated body weight loss and significantly restored the colon length and weight. These improvements were particularly notable in the medium- and high-dose groups, indicating a dose-responsive therapeutic effect against DSS-induced UC. Histopathological assessments confirmed that naringin alleviated colonic tissue damage and maintained mucosal integrity, particularly at higher doses. Treatment with naringin significantly restored mucosal integrity and reduced colon injury in a dose-dependent manner, with the highest dose (100 mg/kg) fully preventing colitis-associated histopathological alterations; specifically, it significantly down-regulated the expression of key proinflammatory cytokines, including TNF-α, IL-6, and IL-1β, in both colonic tissues and the systemic circulation. This suppression of cytokine production was associated with a notable reduction in colonic inflammation and tissue damage. Concurrently, naringin improved the redox balance by attenuating oxidative stress; in particular, the level of MDA—a marker of lipid peroxidation—was significantly decreased, while antioxidant defense compounds, including GSH and SOD, were restored in a dose-dependent manner. These findings highlight the dual role of naringin in both mitigating inflammatory cascades and preserving cellular oxidative homeostasis under DSS-induced colitis. Significantly, naringin enhanced the expression of tight junction proteins, including occludin and ZO-1, indicating its protective role in preserving epithelial barrier function. The compound also down-regulated inflammatory enzymes such as iNOS and COX-2, further supporting its anti-inflammatory properties [146].

Similarly, it has been reported that treatment with naringin (25, 50, or 100 mg/kg, orally for 7 days) alleviated colonic pathological damage, colon length shortening, and the DAI in DSS-induced ulcerative colitis mice. In addition, this compound activated PPAR-γ and consequently inhibited NF-κB activation; suppressed the activation of the NLRP3 inflammasome and MAPK; and regulated ZO-1 expression, favoring maintenance of the intestinal architecture. These results suggest that this compound is a potent candidate for preserving the health of mice with ulcerative colitis [143].

In 2024, Wang et al. [147] reported that the oral administration of hesperetin (100 mg/kg) daily for 7 days improved body weight, colon length, and the DAI; reduced serum levels of IL-6 and TNF-α, the mRNA expression of IL-6, IL-1β, TNFα, MMP9, PTGS2 (prostaglandin-endoperoxide synthase 2), and ACSL4 (Acyl-CoA Synthetase Long Chain Family Member 4), and MDA levels in the colon; and increased the mRNA expression of TP53 and Gpx4 (Glutathione peroxidase 4) in the colon of mice with DSS-induced ulcerative colitis, suggesting that this flavonoid exhibits anti-colitic activity in these animals by inhibiting ferroptosis through modulation of some of the abovementioned indicators, such as PTGS2, ACSL4, and Gpx4. Therefore, hesperetin may be considered as a potentially beneficial adjunct in colitis therapy. Also in 2024, Chi et al. [148] reported that the oral administration of L-arginine-induced self-assembled hesperetin nanoparticles (100 mg/Kg) daily for 21 days improved body weight, colon length, and the DAI; reduced MDA, IL-6, IL-1β, and TNF-α levels, as well as MPO activity; and increased GSH, CAT, SOD, and IL-10 levels and the expression of ZO-1, claudin-1, occludin, and Muc-2 in the colon of mice with DSS-induced ulcerative colitis. Furthermore, they demonstrated that hesperetin nanoparticles are stable in the gastrointestinal tract, effectively target the inflamed colon, and have good biocompatibility, tolerance, and safety in mice, as no damage to kidney and liver function or histological alterations in major organs were observed. Finally, treatment with hesperetin nanoparticles yielded superior results for all of the colitis-related parameters evaluated, when compared to the first-line drug (5-aminosalicylic acid). The authors concluded that the successfully prepared L-arginine-induced self-assembled hesperetin nanoparticles are an excellent candidate for the treatment of colitis, thanks to the combination of their manufacturing characteristics and the biomedical properties of this flavonoid. Zhang et al. have reported that treatment with hesperetin (20 mg/kg, intraperitoneal injection for 7 days) decreased colon length shortening, weight loss, histopathological scores, and the DAI in DSS-induced ulcerative colitis mice. Furthermore, this compound increased MUC2, occludin, and ZO-1 protein expression; reduced high mobility group protein 1 (HMGB1), IL-6, IL-18, IL-1β, and TNF-α; suppressed MLKL and RIPK3 expression; and inhibited RIPK3/MLKL necroptosis signaling. These results suggest that this compound ameliorates colitis and protects the epithelial barrier through inhibition of intestinal necroptosis [149].

In another study, hesperidin treatment (10, 20, or 40 mg/kg, orally for 13 days) attenuated body weight loss, the DAI, colon length shortening, and histopathological scores in DSS-induced ulcerative colitis mice. Moreover, this compound reduced MDA activity; increased GSH and SOD activities; up-regulated the Nrf2 pathway; raised NQO1 and HO-1 protein expression; decreased TNF-α and IL-6 levels and MPO activity; raised IL-10 levels in colonic tissues; increased ZO-1 and occludin protein expression; and up-regulated the Treg cell population in the colon. These results suggest that hesperidin exerts effects on colitis by ameliorating the Nrf2 pathway, maintaining the function of the intestinal barrier, and increasing the population of Treg cells [150]. Similarly, it has been reported that treatment with hesperidin (50 mg/kg via gastric gavage for 14 days) decreased colon length shortening, rectal bleeding, diarrhea, and body weight loss in DSS-induced ulcerative colitis in rats. This compound also reduced SPHK1 levels, sphingosine phosphate phosphatase 2 (SGPP2) mRNA, and MIP-1α levels in the colon; increased Bcl-2 expression; increased peroxisome proliferator-activated receptor-gamma-coactivator 1-α (PGC1α) colonic levels, thus ameliorating mitochondrial biogenesis; decreased peroxynitrite and nitric oxide levels; and raised SOD levels. These results indicate hesperidin as a potential candidate for the treatment of colitis [151].

**Table 8:** Effects of flavanones in different models of DSS-induced colitis.

Flavanone	Model	Properties	Ref.
Pinocembrin	DSS/rat	Improves clinical symptoms, intestinal goblet cell loss, and colonic pathological damages; inhibits TNF-α, IL-6, and IL-1β gene expression; raises occludin and ZO-1 protein gene expression.	[134]
	DSS/mice	Alleviates the severity of colitis; decreases body weight loss, blood in stool, diarrhea, and colon shortening; decreases TLR4 protein level and phosphorylation of p65; suppresses Myd88, TLR4, iNOS, TNF-α, and COX-2 mRNA expression levels.	[135]
Eriocitrin		Decreases colon histopathological damage; colon shortening; body weight loss; secretion of TNF-α, IL-1β, IL-6, and MPO; and the accumulation of infiltrated inflammatory cells.	[136]
Naringenin		Alleviates body weight loss; reduces the DAI; mitigates colon shortening; activates the Nrf2 pathway and inhibits proinflammatory markers such as NF-κB, TNF-α, and IL-1β.	[137]
		Decreases body weight loss and TNF-α-positive epithelial cells; inhibits TNF-α mRNA expression; increases junctional adhesion molecule-A and claudin-3, as well as CD206 and IL-10 mRNA expression; stimulates polarization of M2-type macrophages.	[138]
		Decreases colon shortening, the DAI, and gene expression of IL-17A, IL-6, and MIP-2; increases expression of claudin-3, junctional adhesion molecule-A, and occludin.	[139]
		Down-regulates MCP-1, ICAM-1, iNOS, COX-2, IL-6, and TNF-α mRNA; decreases IL-6 and TNF-α, and TLR4 mRNA and protein; reduces p-NF-κB (p65) protein levels and p-IκBα protein.	[140]
Naringin		MC@Nar-NPs reduce the DAI; mitigate body weight loss; preserve colon length; restore goblet cells; decrease IL-6, IL-1β, TNF-α, and ROS levels; increase SOD, CAT, and GSH activities and ZO-1 expression.	[141]
		Mitigates body weight loss; restores colon length and weight; reduces the DAI, TNF-α, IL-6, IL-1β, iNOS, COX-2, and MDA levels; increases GSH and SOD activity and occludin and ZO-1 expression.	[146]
		Alleviates colonic pathological damage, colon length shortening, and the DAI; activates PPAR-γ; inhibits NF-κB activation; suppresses the activation of NLRP3 inflammasome and MAPK; regulates ZO-1 expression.	[143]
Hesperetin		Improves mouse body weight and colon length; reduces the DAI; decreased serum IL-6 and TNF-α levels, and mRNA expression of IL-6, IL-1β, TNFα, MMP9, PTGS2, ACSL4, and MDA levels in the colon; increases mRNA expression of TP53 and Gpx4 in the colon.	[147]
		Improves mouse body weight and colon length; reduces the DAI, MDA, IL-6, IL-1β, and TNFα levels and MPO activity; increases GSH, CAT, SOD, and IL-10 levels and the expression of ZO-1, claudin-1, occludin, and *Muc-2* in the colon.	[148]
		Decreases colon length shortening, weight loss, histopathological scores, and the DAI; raises MUC2, occludin, and ZO-1 protein expression; reduces HMGB1, IL-6, IL-18, IL-1β, and TNF-α; suppresses MLKL and RIPK3 expression; inhibits RIPK3/MLKL necroptosis signaling.	[149]
Hesperidin		Attenuates body weight loss, the DAI, colon length shortening, and histopathological scores; reduces MDA activity; increases GSH and SOD activities and ZO-1 and occludin protein expression; up-regulates Nrf2 pathway and Treg cells; raises NQO1 and HO-1 protein expression and IL-10 levels; decreases TNF-α and IL-6 levels and MPO activity.	[150]
	DSS/rat	Decreases colon length shortening, rectal bleeding, diarrhea, body weight loss, and peroxynitrite and nitric oxide levels; reduces SPHK1 levels, SGPP2 mRNA, and MIP-1α level; increases Bcl-2 expression; raises PGC1α and SOD levels.	[151]

Taken together, the studies summarized in this section indicate that flavanones may act through interconnected antioxidant, inflammatory, immune, and epithelial barrier pathways. Several findings support the existence of cross-talk between redox regulation and inflammatory signaling; in particular, naringenin increased Nrf2, HO-1, and NQO1 expression while reducing NF-κB, TNF-α, and IL-1β, and the partial reversal of these effects, mediated by the Nrf2 inhibitor ML385, provides experimental support for a functional role of Nrf2 in this protective activity [137]. Similarly, hesperidin was associated with activation of the Nrf2/NQO1/HO-1 pathway together with reduced inflammatory mediators and improved intestinal barrier markers [150]. Another relevant regulatory node is PPAR-γ, which was associated with inhibition of NF-κB activation in naringin-treated colitis models [128,143]. Naringin was also reported to suppress NLRP3 inflammasome and MAPK activation, suggesting that PPAR-γ, NF-κB, NLRP3, and MAPK may represent interconnected inflammatory nodes involved in its protective effects [143]. In parallel, pinocembrin and naringenin were reported to suppress the TLR4/MyD88/NF-κB axis, providing an example of an upstream innate immune pathway converging on NF-κB-mediated inflammatory signaling [135,140]. Hesperetin additionally modulated the JAK2/STAT3/SOCS3 pathway, suggesting the regulation of cytokine-dependent signaling through a mechanism distinct from—but potentially complementary to—NF-κB modulation [132]. Finally, the reported effects of hesperetin on ACSL4, MDA, and GPX4 suggest the modulation of ferroptosis-related processes [147]. Overall, these findings support a model in which flavanones can simultaneously influence redox homeostasis, inflammatory signaling, immune responses, and epithelial barrier integrity. However, except for the experimental evidence supporting the involvement of Nrf2 in naringenin-mediated protection [137], most studies have only demonstrated pathway modulation rather than a direct molecular interaction with the reported targets. Therefore, while the antioxidant effects may contribute to the regulation of inflammatory pathways, the available evidence does not allow these mechanisms to be collectively interpreted as a single linear signaling cascade.

Flavanones, such as hesperetin, naringenin, naringin, hesperidin, pinocembrin, and eriocitrin, have been shown to be effective in mitigating colitis due to their anti-inflammatory and antioxidant properties. Their mechanisms of action involve reductions in the levels of proinflammatory cytokines (TNF-α, IL-1β, IL-6), modulation of crucial inflammatory signaling pathways (NF-κB, JAK2/STAT3/SOCS3, PPAR-γ, NLRP3 inflammasome), and improvement of intestinal barrier integrity through increases in tight junction proteins (ZO-1, occludin, claudin). They also contribute to protection against oxidative damage and restoration of cellular balance. Furthermore, nanoparticle formulations of different flavanones have been shown to significantly improve their bioavailability and therapeutic efficacy, offering a very promising strategy for the development of advanced therapies.

## 5. Isoflavones and Flavan-3-Ols with Activity Against Ulcerative Colitis

In one study, it was reported that the oral administration of genistein (25 mg/kg) daily for 14 days improved colon length and weight; reduced MDA levels and the expression of caspase-9, caspase-8, and caspase-3, as well as the expression and protein levels of BAX; and increased the expression of TFAM (mitochondrial transcription factor A), PGC-1 (peroxisome proliferator-activated receptor-gamma coactivator), HO-1, Nrf2, and Bcl-2 genes, as well as their protein levels, in the colon of rats with acetic-acid-induced ulcerative colitis. Therefore, the authors suggested that this flavonoid exerts effects on animal colitis—at least in part—by improving various parameters associated with antioxidant activity and mitochondrial biogenesis. Furthermore, they mentioned that the tested dose could be achieved by consuming between 200 and 100 g of soybeans in the diet, making genistein a relevant and accessible candidate for the treatment of this disease [152] (Table 9). Another similar study reported that the oral administration of genistein (100 mg/kg) daily for 14 days improved the DAI and colon weight/length ratio, increased goblet cell numbers and SOD activity, and reduced INF-γ production; IL-6, IL-1β, TNF-α, IRF-1, NO, COX-2, and MPO levels; and STAT1, STAT3, JAK1, JAK2, iNOS, NF-κB, and caspase-3 expression in rats with acetic-acid-induced ulcerative colitis. These findings suggest that this flavonoid may exert effects on colitis in these model animals by modulating multiple inflammatory pathways, including the IFN-γ/JAK1/STAT1 pathway, the IRF-1/iNOS/NO axis, and the JAK2/STAT3/COX-2 pathway. Furthermore, combined treatment of genistein with the drug sulfasalazine was assessed, which showed the best results. Therefore, implementing combined treatments could offer greater benefits for the treatment of colitis [153]. Another study reported that genistein (10 mg/kg), administered orally via gavage for 14 days increased the colon length and body weight and decreased the inflammation score in DSS-induced ulcerative colitis mice, thereby reducing the severity of colitis. In addition, this treatment decreased the serum levels of IL-1β, TNFα, MCP-1, and IL-6. These results indicate that this compound could be used to ameliorate the symptoms of and/or treat colitis [154]. In 2024, Jia et al. found that the administration of genistein (40 or 20 mg/kg) intragastrically daily for 10 days improved the DAI, body weight, and colon length; increased MUC2 and IL-10 levels and the expression of occludin, ZO-1, and M2 macrophage markers (CHI3L3 and Arg-1); and reduced TNF-α, IL-1β, and IL-6 levels, MPO activity, and the expression of M1 macrophage markers (TNF-α and iNOS) in mice with DSS-induced ulcerative colitis. Furthermore, the effects of this flavonoid were dose-dependent, with the highest dose tested showing the best results. Therefore, the authors concluded that genistein may exert its effects on colitis in the model animals—at least in part—through its anti-inflammatory activity and the polarization of M1 to M2 macrophages. As such, this flavonoid has promising potential as a candidate for the treatment of UC [155].

In one investigation, administration of daidzein (10 mg/kg orally for seven days) ameliorated colon length shortening, the ulcer area, and the DAI in DSS-induced ulcerative colitis mice. Additionally, this compound reduced the MPO level; suppressed p-IKK, p-IκB-α, and NF-κB (p-65) expression; and decreased IL-6, IL-1β, and TNF-α levels in the colon. These results suggest that daidzein has inflammatory activity and may be a considered as a candidate for the treatment of colitis [156].

One study reported that the administration of epigallocatechin-3-gallate (EGCG) (50 or 100 mg/kg via gavage for seven days) reduced the DAI and spleen index, while also mitigating the erosion of colon tissue, in DSS-induced ulcerative colitis mice. Furthermore, this compound decreased IL-17 and IL-6 secretion, modulated the mouse splenic Treg/Th17 cell ratio, and raised TGF-β1 and IL-10 plasma levels, while reducing STAT3 and HIF-1α protein expression in the colon. These results suggest that EGCG ameliorates colitis through the suppression of IL-6 secretion and modulation of the Treg/Th17 equilibrium [157]. Another study reported that EGCG administration (20 or 50 mg/kg via oral gavage for 7 days) improved the DAI and body weight loss in DSS-induced ulcerative colitis mice. Additionally, this compound prevented colon shortening; ameliorated histopathological and intestinal permeability changes; mitigated colon inflammation by inhibiting MCP-1, TNF-α, and IL-6 levels in the colon; and suppressed CD68^+^ macrophage and CD3^+^ T cell infiltration. These results suggest that this compound effectively alleviates colitis, as it decreases intestinal permeability and inflammation [158].

Wu et al. [159] tested two different administration routes for EGCG treatment: oral and rectal. They induced experimental colitis with DSS in mice for 7 days, after which EGCG was administered via either route for 3 days at a dose of 50 mg/kg of body weight. Their results demonstrated that oral administration was more effective than rectal administration for all the parameters evaluated, leading to superior reduction in the DAI, attenuation of body weight loss, and colon length improvement. Furthermore, the plasma and colonic levels of proinflammatory cytokines (e.g., IL-1β, IL-6, IL-8, and TNF-α) were significantly decreased in mice treated with oral EGCG compared to those treated rectally, where EGCG failed to attenuate inflammation or the intestinal pathology in the latter. Similarly, oral EGCG decreased levels of certain oxidative stress parameters, such as MPO and MDA, and increased the activity of antioxidant enzymes including SOD and CAT. These results suggest that it is essential to consider the route of administration, as different factors (such as the microbiota) may affect compounds when administered orally.

Research on the treatment of colitis with EGCG has not only focused on the route of administration, but also on the use of different materials to improve its bioavailability. Liu et al. combined EGCG with cathelicidin-BF (CBF), an antimicrobial peptide, and encapsulated both in nanoparticles made with Bombyx mori silk fibroin and coated with an alginate and chitosan gel for protection during passage through the upper gastrointestinal tract. Subsequently, the EGCG nanoparticles were administered to mice with DSS-induced colitis at a concentration of 0.5 mg/kg orally for 7 days. Their results demonstrated the excellent biosafety of treatment with the EGCG nanoparticles, as the concentrations of hepatotoxicity and nephrotoxicity biomarkers were comparable to those in the healthy controls. Furthermore, the administration of EGCG nanoparticles improved the body weight and colon length; increased IL-10 levels and the expression of MUC2, ZO-1, and occludin; and decreased MPO activity and the levels of TNF-α and IL-12 in the colitic mice. The authors concluded that EGCG nanoparticles exert efficient therapeutic effects, and can be considered as part of a comprehensive strategy for the treatment of UC [160]. Other authors have formed an inclusion complex of γ-cyclodextrin (γ-CD) with EGCG to study its effects on UC. The γ-CD–EGCG complex (50 mg/kg) was administered orally for 9 days to mice with DSS-induced colitis. Treatment with this complex improved the body weight and colon length in the mice, increased GSH levels, and decreased the DAI, MPO activity, and TNF-α, IL-1β, and IL-6 levels in the colon. The authors concluded that the safety of long-term administration of the γ-CD–EGCG complex, along with its potential for industrial-scale production, positions it as a promising formulation for use in the treatment of UC [161].

Similarly, Diwan et al. verified the effectiveness of EGCG in a DSS-induced model of experimental colitis where, unlike in the previously described investigations, EGCG was administered for 30 days at the chosen dose (100 mg/kg/day) before DSS-mediated induction of colitis for 6 days. Pre-treatment with EGCG improved body weight and colon shortening and decreased the DAI, MDA activity, and levels of the proinflammatory cytokines IL-1β, IL-6, and TNF-α. Another important finding was that the mRNA expression of Mucin-2 (a mucus secretory protein), occludin, and ZO-1 was increased in the mice pre-treated with EGCG. The authors concluded that the use of this flavonoid in a murine model of colitis could significantly attenuate inflammatory damage, and may even improve the architecture and functional homeostasis of intestinal epithelial cells [162].

In one of the most recent studies, conducted by an Asian team in 2025, the presented results were similar to those reported in various previous publications [159,160,161,162]. In this research, mice were therapeutically administered EGCG (50 mg/kg) for 28 days.

After this, they were administered a DSS solution for 6 days, yielding results that demonstrate the effectiveness of EGCG. First, EGCG treatment led to improvements in body weight and colon shortening, as well as a reduction in the DAI. In this research, an inflammatory model was established using RSL3, which causes a type of cell death known as ferroptosis that is associated with iron-dependent lipid peroxidation, resulting in reduced cell viability and increased ferroptosis markers. This has become a widely studied mechanism in research on degenerative and inflammatory diseases. In this model, EGCG supplementation effectively suppressed inflammatory markers such as IL-1β, IL-6, and TNF-α; decreased the expression of ACSL4 (a critical enzyme in lipid peroxidation and ferroptosis) and MDA levels; increased the GSH content and gene expression of different ferroptosis-regulatory proteins such as GPX4, SLC7A11, and SLC3A2; and positively modulated the Nrf2 signaling pathway (a key antioxidant defense system), thus restoring the redox balance and iron metabolism. These results suggest that EGCG has promising potential as a ferroptosis inhibitor and as a therapeutic agent for colitis, mediated by activation of the Nrf2–GPX4 pathway [163].

Another investigation studied the protective activity of EGCG in a rat model of DSS-induced ulcerative colitis and explored the functions of the TLR4/MyD88/NF-κB signaling pathway. The EGCG treatment (50 mg/kg, intraperitoneally for 15 days) ameliorated intestinal mucosal damage, decreased serum IFN-γ and IL-2 levels, and increased IL-10 and IL-4 levels. Additionally, it maintained the Th1/Th2 equilibrium and decreased the expression of NF-κB, MyD88, and TLR4. These results suggest that EGCG can suppress the intestinal inflammatory response, thus decreasing the severity of disease, by maintaining the Th1/Th2 equilibrium through the TLR4/MyD88/NF-κB signaling pathway [164].

**Table 9:** Effects of isoflavones and flavan-3-ols in different models of colitis induction.

Isoflavone/ Flavan-3-Ol	Model	Properties	Ref.
Genistein	Acetic acid/rats	Improves colon length and weight; reduces MDA levels, the expression of caspase-9, caspase-8, and caspase-3, and the expression and protein levels of BAX; increases the expression of TFAM, PGC-1, HO-1, Nrf2, and Bcl-2 genes, as well as their protein levels in the colon.	[152]
		Improves the DAI and colon weight/length ratio; increases goblet cell number and SOD activity; reduces INF-γ production, IL-6, IL-1β, TNF-α, IRF-1, NO, COX-2, and MPO levels, as well as STAT1, STAT3, JAK1, JAK2, iNOS, NF-κB, and caspase-3 expression; potentially modulates the INF-γ/JAK1/STAT1 pathway, the IRF-1/iNOS/NO axis, and the JAK2/STAT3/COX-2 pathway.	[153]
	DSS/mice	Raises colon length and body weight; decreases the inflammation score and IL-1β, TNFα, MCP-1, and IL-6 serum levels.	[154]
		Improves the DAI, body weight, and colon length; increases MUC2 and IL-10 levels, as well as the expression of occludin, ZO-1, CHI3L3, and Arg-1; reduces TNF-α, IL-1β, and IL-6 levels, MPO activity, and the expression of TNF-α and iNOS.	[155]
Daidzein		Ameliorates colon length shortening, ulcer area, and the DAI; reduces MPO level; suppresses p-IKK, p-IκB-α, and NF-κB (p-65) expression and IL-6, IL-1β, and TNF-α levels.	[156]
EGCG		Reduces the DAI and STAT3 and HIF-1α protein expression; mitigates erosion of colon tissue; decreases IL-17 and IL-6 secretion; modulates the mouse splenic Treg/Th17 cell ratio; raises TGF-β1 and IL-10 plasma levels.	[157]
		Ameliorates the DAI, body weight loss, histopathological changes, and intestinal permeability; avoids colon shortening; inhibits MCP-1, TNF-α, and IL-6 levels; suppresses CD68^+^ macrophage and CD3^+^ T cell infiltration.	[158]
		Oral administration reduces the DAI; attenuates body weight loss; improves colon length; decreases IL-1β, IL-6, IL-8, TNF-α, MPO, and MDA levels; increases SOD and CAT activities in plasma and colonic tissue.	[159]
		EGCG nanoparticles improve body weight and colon length; increase IL-10 levels and the expression of MUC2, ZO-1, and occludin; decrease MPO activity and the levels of TNF-α and IL-12.	[160]
		γ-CD–EGCG complex improves body weight and colon length; decreases the DAI, MPO activity, and levels of TNF-α, IL-1β, and IL-6; increases GSH levels.	[161]
		Improves body weight and colon shortening; decreases DAI, MDA activity, and IL-1β, IL-6, and TNF-α levels; increases the mRNA expression of Mucin-2, occludin, and ZO-1.	[162]
		Pre-administration of EGCG improves body weight and colon shortening; reduces DAI; suppresses IL-1β, IL-6, and TNF-α; decreases expression of ACSL4 and MDA levels; increases GSH content and gene expression of GPX4, SLC7A11, and SLC3A2; positively modulates Nrf2 signaling pathway.	[163]
	DSS/rat	Ameliorates intestinal mucosal damage; decreases serum IFN-γ and IL-2 levels; raises IL-10 and IL-4 levels; maintains the Th1/Th2 equilibrium; decreases NF-κB, MyD88, and TLR4 expression.	[164]

Taken together, the findings summarized in this section suggest that isoflavones and flavan-3-ols may modulate ulcerative colitis through interconnected antioxidant, inflammatory, immune, and epithelial barrier pathways, rather than through a single molecular target. Genistein provides evidence of cross-talk between redox regulation and inflammatory signaling, as it increased Nrf2 and HO-1 expression while reducing oxidative stress and inflammatory mediators [152], and was also associated with modulation of the IFN-γ/JAK1/STAT1 and JAK2/STAT3/COX-2 pathways [153]. Similarly, EGCG modulated STAT3 in association with changes in the Treg/Th17 balance [157]. NF-κB represents another important inflammatory node in this network, as genistein and daidzein reduced NF-κB-related signaling [153,156], whereas EGCG was reported to suppress the TLR4/MyD88/NF-κB pathway [164], providing a potential link between upstream innate immune recognition and downstream inflammatory cytokine production. In addition, the effects of EGCG on oxidative stress and ferroptosis provide a further connection between redox homeostasis and inflammation. EGCG increased Nrf2 signaling and the expression of GPX4, SLC7A11, and SLC3A2; increased GSH; and reduced MDA and ACSL4 expression [163], suggesting that the modulation of antioxidant and ferroptosis-related pathways may contribute to its protective effects. These signaling events may ultimately converge on the preservation of intestinal homeostasis, as several studies reported simultaneous reductions in inflammatory mediators and improvements in MUC2, occludin, and ZO-1 expression [160,162]. Nevertheless, the available studies generally investigated individual signaling components, precluding the establishment of a single linear cascade connecting Nrf2, NF-κB, JAK/STAT, and other pathways. Moreover, although pathway modulation effects were demonstrated, the direct interactions of these flavonoids with their corresponding molecular targets have generally not been established. Therefore, their effects may involve both the specific modulation of signaling pathways and indirect mechanisms associated with cellular redox status improvements.

Despite their therapeutic potential, the administration of flavonoids such as EGCG poses several safety-related challenges. Exposure to high doses of EGCG—particularly when administered as concentrated preparations and under fasting conditions—has been associated with liver injury, and safety assessments have reported increased liver transaminases following consumption of EGCG at doses ≥ 800 mg/day from supplements [165,166].

Isoflavones (e.g., genistein and daidzein) and flavan-3-ols (e.g., EGCG) have been shown to exhibit significant therapeutic effects in animal models of colitis. These compounds act as anti-inflammatory and antioxidant agents, reducing tissue damage, inflammatory cell infiltration, and proinflammatory cytokine production. They may also enhance intestinal barrier integrity and modulate immune responses, including the balance between Th1/Th2 and Treg/Th17 cells, as well as macrophage polarization. Recent research has further emphasized that consideration of the route of administration (e.g., oral versus rectal for EGCG) and the use of nanoparticle systems (EGCG/CBF) are crucial for optimizing their bioavailability and efficacy, allowing for more effective delivery to the site of inflammation and maximization of their benefits in alleviating the symptoms of colitis and restoring intestinal homeostasis.

Flavonoids exhibit a wide range of biological activities that may counteract the pathological alterations associated with ulcerative colitis. To provide a clearer and more comprehensive overview of the findings discussed in this review, Figure 1 summarizes the main beneficial effects of flavonoids at the intestinal level.

## 6. Limitations and Future Perspectives

Despite the promising findings summarized in this review, several limitations should be considered when interpreting the available evidence. First, most of the studies evaluating the effects of propolis and its flavonoids against ulcerative colitis have utilized experimental animal models, particularly chemically induced models using DSS, TNBS, or acetic acid. Although these models reproduce several relevant features of intestinal inflammation, they do not fully recapitulate the complex and multifactorial pathophysiology of human ulcerative colitis. Second, substantial heterogeneity exists among studies regarding the used animal species and strains; colitis induction protocol; dose, route, and duration of treatment; propolis origin; classes of flavonoids; and experimental endpoints. This heterogeneity limits direct comparisons among studies and prevents the establishment of standardized therapeutic doses.

Another important limitation is the considerable chemical variability of propolis, as its composition depends on its botanical and geographical origin, season of collection, and bee species. Consequently, propolis extracts obtained from different sources cannot necessarily be considered chemically equivalent. Moreover, in most studies, the concentrations of individual flavonoids in the administered propolis extracts have not been sufficiently characterized to allow for direct comparisons with effective doses of purified compounds. Therefore, future studies should combine comprehensive chemical profiling with quantitative determination of individual flavonoids and other bioactive constituents, in order to establish more accurate relationships among composition, dose, bioavailability, and biological activity variables.

From an evidence-based medicine perspective, an additional limitation is the scarcity of information regarding the pharmacokinetics, bioavailability, long-term safety, toxicity, and potential interactions of propolis and its individual components with conventional treatments for ulcerative colitis. The effects observed in animal models should therefore not be directly extrapolated to clinical efficacy in humans.

Future research perspectives should prioritize the development of standardized propolis preparations, reproducible characterization methods, dose–response studies, and comparative investigations of whole propolis, flavonoid-enriched fractions, and purified compounds. Further characterization of relevant molecular mechanisms, including the interactions among inflammatory, antioxidant, intestinal barrier, immune, and microbiota-related pathways, may also help in identifying the compounds or combinations of compounds that contribute most to the observed effects.

Importantly, as a future perspective, translation into clinical practice will require well-designed human studies assessing efficacy, safety, tolerability, pharmacokinetics, and interactions with established ulcerative colitis therapies. In this regard, randomized controlled trials utilizing adequately characterized propolis preparations and clearly defined clinical and biochemical endpoints would be particularly valuable. Such studies could determine whether the beneficial effects observed in experimental models can be reproduced in patients, and whether certain types of propolis or flavonoids identified in their chemical composition could serve as part of complementary interventions. Although the current evidence supports propolis and its flavonoids as promising sources of bioactive compounds for further investigation, their clinical utility for ulcerative colitis remains to be established.

## 7. Conclusions

UC is a chronic, immune-mediated inflammatory disorder with a growing incidence worldwide and a significant economic burden, and the current first-line treatments for this condition are often associated with adverse effects. Research conducted over the past 15 years has extensively highlighted the promising potential of flavonoids as alternative or complementary therapeutic options for UC, thanks to their diverse beneficial properties.

Flavonoids (flavones, flavonols, flavanones, isoflavones, and flavan-3-ols) derived from bee products exert protective effects against colitis through multiple interconnected mechanisms. First, they exhibit potent anti-inflammatory activity, significantly reducing proinflammatory cytokine levels, modulating various key signaling pathways (e.g., NF-κB, MAPK, JAK/STAT, PI3K/AKT, TLR4/MyD88, and NLRP3 inflammasome pathways), and influencing macrophage polarization and T cell balance (Th17/Treg). They also show antioxidant properties, as evidenced by increases in endogenous antioxidant levels and reductions in markers of oxidative stress. Furthermore, they enhance intestinal barrier function by strengthening the integrity of the intestinal epithelium, which is evidenced by increased expression of tight junction proteins, improved goblet cell function, and enhanced mucin secretion. Finally, they alleviate the clinical symptoms of colitis, particularly attenuating weight loss, colonic shortening, histopathological damage, and the DAI.

Recent research has also emphasized the importance of advanced formulations such as nanoparticles, which improve the solubility, stability, bioavailability, and targeted delivery of flavonoids to the inflamed colon. These technological advances further enhance the therapeutic effects of flavonoids, making them very promising candidates for the development of complementary or alternative treatments for UC.

Further research is still needed regarding human pharmacokinetics, dose escalation, randomized controlled trials, and long-term safety, in order to provide more precise information on the appropriate use of propolis and flavonoids in humans; nevertheless, the evidence currently available from preclinical studies is promising.

## Figures and Tables

**Figure 1:**
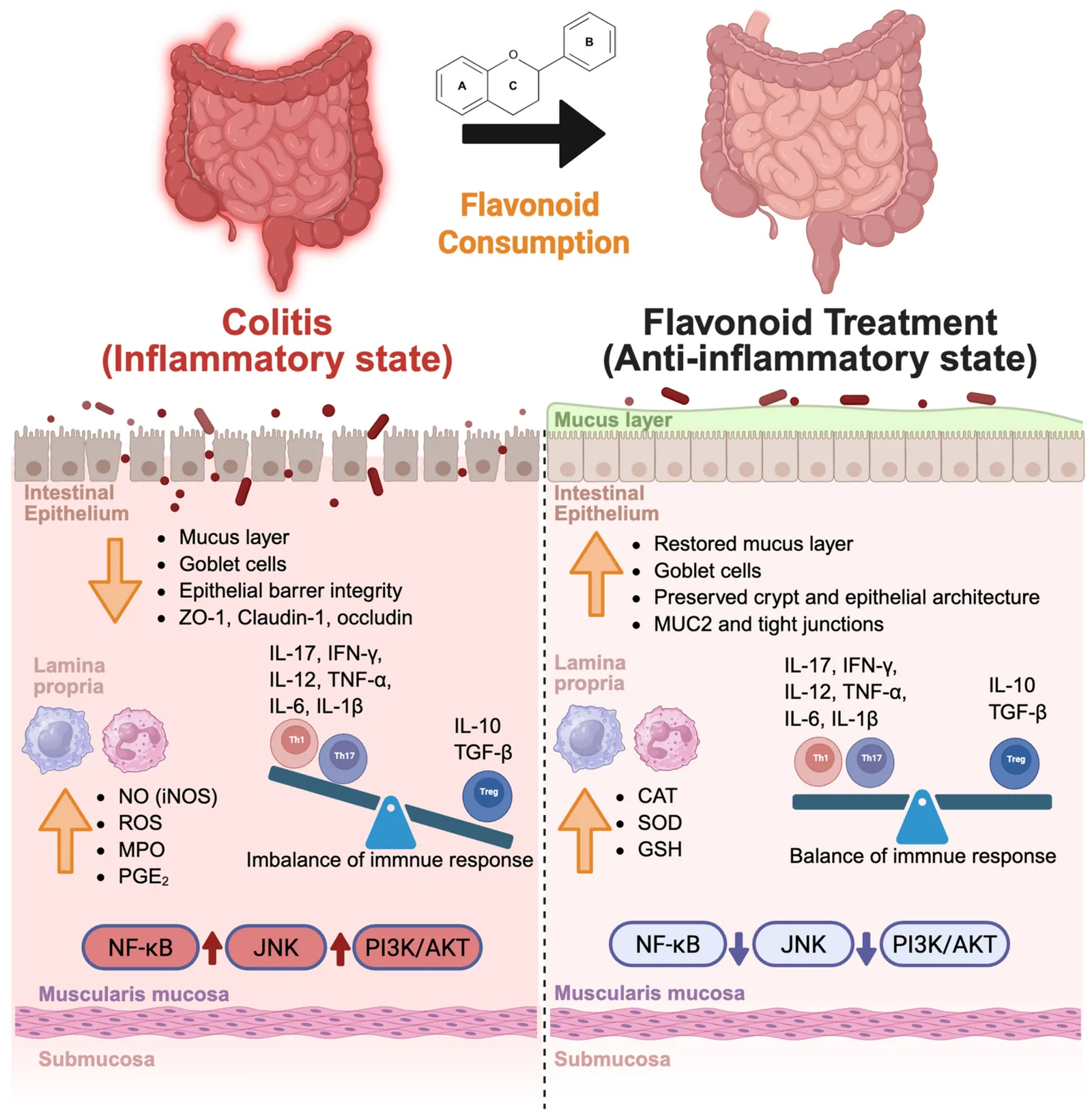
Flavonoids identified in propolis exert multiple beneficial effects at the intestinal level that may contribute to ameliorating ulcerative colitis. These effects include restoration and maintenance of the intestinal mucus layer, thereby limiting direct contact between the gut microbiota and the intestinal epithelium, preventing microbial invasion. Flavonoids also reduce the infiltration of inflammatory cells, including macrophages, neutrophils, and lymphocytes. In parallel, they modulate Th17, Th1, and Treg immune responses; decrease the levels of inflammatory mediators, including MPO and PGE_2_, as well as reactive oxygen and nitrogen species; enhance the levels of antioxidant defense compounds, including SOD, CAT, and GSH; and modulate inflammation-related signaling pathways, such as NF-κB, JNK, and PI3K/AKT.

**Table 1:** Examples of compounds from each group of flavonoids.

Flavonoid Group	Example(s)
Flavones	Acacetin, apigenin, luteolin, baicalin, baicalein, tangeretin
Flavonols	Quercetin, galangin, kaempferol, rutin, fisetin, myricetin, myricitrin
Flavanones	Pinocembrin, naringenin, naringin hesperidin, hesperetin, eriocitrin
Isoflavones	Genistein, daidzein
Flavan-3-ols	Epigallocatechin-3-gallate (EGCG)

**Table 2:** Effects of various flavones in different models of colitis induction.

Flavone	Model	Properties	Ref.
Apigenin	Acetic acid/rats	Improves body weight and colon length; increases GSH levels; reduces DAI score, MDA, MAdCAM-1, NOx, and IL-1β levels, and MPO activity in the colon.	[45]
Tangeretin	TNBS/mice	Inhibits MPO activity and MAPK and NF-κB activation; raises expression of ZO-1, claudin-1, and occludin, the differentiation of regulatory T cells, and the expression of IL-10 and Foxp3; suppresses differentiation of Th17 and Th1 cells and the expression of TNF-α, IL-12, IL-17, IFN-γ, RORγt, and T-bet.	[46]
Baicalin	TNBS/rats	Down-regulates the quantity of macrophages and the expression of MIF, MCP-1, and MIP-3α.	[47]
		Decreases DAI; mitigates mucosal damage; up-regulates the Th17/Treg ratio; increases MUC2, occludin, and ZO-1 protein expression and SOD and GSH levels; inhibits MDA and ROS levels.	[48]
		Decreases DAI; ameliorates shortening of the colon and weight loss; reduces IL-1β and TNF-α levels, IFN-γ and IL-12, and MPO activity; down-regulates the quantity of Th17 cells and the levels of IL-6, IL-17, and RORγt; increases the number of Treg cells and the levels of Foxp3, IL-10, and TGF-β.	[49]
		Decreases IL-1β, TNF-α, and IL-6 secretion; raises the IL-10 level; stimulates ZO-1 protein expression; suppresses the PI3K/AKT signaling pathway.	[50]
		Raises GSH, CAT, and SOD activities; reduces TNF-α, IL-1β, PGE_2_, MPO, and MDA contents, as well as cyt-c, cleaved-caspase9, cleaved-caspase3, NF-κB (p-65), p-IκBα/IκBα, and p-IKKβ/IKKβ expression.	[51]
		Decreases IL-6, TNF-α, and IL-1β production; reduces mucosal TLR4 protein expression, p-NF-κB (p65) protein expression, and p-IκB protein.	[52]

**Table 3:** Effects of various flavones on DSS-induced colitis.

Flavone	Model	Properties	Ref.
Tangeretin	DSS/mice	Mitigates damage in colon tissue, the DAI, and colon length shortening; down-regulates IL-6, IL-1β, and TNF-α; ameliorates ZO-1 and claudin-1 expression.	[53]
Acacetin		Decreases diarrhea, colon shortening, histological damage, body weight loss, and inflammatory infiltration; reduces infiltration of macrophages expressing F4/80 and the levels of iNOS, COX-2, TNF-α, IL-1β, and IL-6 mRNA.	[54]
Apigenin		Ameliorates colon shortening, body weight loss, histological score, and IL-1β levels; regulates the NLRP6 signaling pathway.	[55]
		Improves weight loss; decreases the DAI score and levels of IL-1β, IL-6, and TNF-α; increases IL-10 levels, MPO activity, and the expression of occludin, claudin-1, and ZO-1 in a dose-dependent manner.	[56]
		Reduces the macroscopic and microscopic effects of colitis, serum MMP-3 levels, and IL-18, IL-1β, and TNF-α levels, as a result of the modulation of cleaved caspase-1 and caspase-11 enzymes; down-regulates COX-2 and iNOS expression.	[57]
Baicalein		Improves body weight and colon length; reduces the DAI; increases colonic occludin and ZO-1 levels; modulates the AhR/IL-22 pathway.	[58]
		Decreases body weight loss in a dose-dependent manner, diarrhea, rectal bleeding, colonic ulceration, colon length shortening, the number of CD11b^+^ cells, and TNF-α, IL-1β, and IL-6 levels; mitigates expression of the transcription factor RORγt; suppresses p-STAT3, SPHK1, NOD2, and S1PR1 expression.	[59]
Baicalin		Reduces the DAI; increases the mRNA expression of IL-10; decreases the mRNA expression of IL-23 and TNF-α.	[60]
		Improves mouse body weight and colon length; reduces the DAI and levels of IL-1β, IL-6, TNF-α, and MDA; increases the levels of IL-10, SOD, claudin-1, occludin, and ZO-1.	[61]
		Improves body weight and colon length; reduces the DAI score and serum levels of TNF-α, IL-1β, and IL-6; increases E-cadherin, ZO-1, claudin-5, claudin-4, IL-18, and MUC2 levels and NLRP6 production; reduces caspase-1 production in colonic tissue.	[62]
	DSS/rats	Improves body weight and colon length; reduces the levels of TNF-α, IL-1β, IL-6, and MPO; increases the levels of ZO-1 and IL-10.	[63]

**Table 4:** Effects of luteolin in different models of DSS-induced colitis.

Flavone	Model	Properties	Ref.
Luteolin	DSS/mice	Improves mouse body weight and colon length; reduces the DAI score; increases the expression of claudin-1, occludin, and ZO-1 in the colon; decreases the levels of IL-1β, IL-6, and TNF-α; increases the production of IL-10.	[66]
			[67]
		Improves histological score and colon length shortening; suppresses IFN-γ-producing CD4^+^ T cells and macrophage infiltration; decreases COX-2, IFN-γ, TNF-α, IL-1β, and IL-6 mRNA expression.	[64]
		Mitigates colon histological damage, DAI, and colon shortening; reduces IL-6, TNF-α, and iNOS expression and MDA content; increases CAT and SOD activities, Nrf2 levels, and its downstream targets NQO1 and HO-1.	[65]
		Improves body weight and colon length; reduces DAI; increases the expression of claudin-1, occludin ZO-1, and GSH; reduces colonic ROS and MPO levels; decreases IL-6, IL-2, IL-17A, IFN-γ, and TNF-α levels; increases IL-10 and IL-4 production in both serum and colonic tissue; increases the numbers of Th2 and Treg cells; decreases the numbers of Th17 and Th1 cells.	[68]
		Attenuates body weight loss, colon length shortening, the DAI, lesion size, and tissue damage; up-regulates ZO-1, occludin, claudin-1, and SHP-1; down-regulates claudin-2, P-STAT-3, and STAT3 (mRNA levels and protein expression).	[69]
		Attenuates body weight loss and colon shortening; raises SOD levels; reduces MDA, TNF-α, IL-1β, and IL-6 levels; decreases HMGB1 protein and mRNA levels and the expression of HMGB1–TLR–NF-κB signaling pathway-related proteins.	[70]
		Improves body weight and colon length; reduces the DAI; increases the expression of occludin, ZO-1, MUC4, MUC2, and MUC1; decreases IL-1β, IL-6, and TNF-α levels; reduces the phosphorylation levels of IKKα/β, IκBα, and p65, thereby decreasing activation of the NF-κB signaling pathway.	[71]
		Improves body weight and colon length; reduces the DAI; increases claudin-1, occludin, and ZO-1 expression and IL-10 production; decreases IL-1β, IL-6, and TNF-α levels; up-regulates the expression levels of CD206 and Arg1; down-regulates the expression level of iNOS; increases activation of the AMPK–PPAR-γ signaling pathway.	[72]
		Decreases body weight loss, the DAI, and colon shortening; increases ERK1/2 phosphorylation; reduces p-p38 and p-JNK1/2 expression, as well as p-STAT3 and p-Akt protein expression.	[73]
		Complete absence of colonic ulceration; reduces the DAI and levels of IL-1β and IL-6.	[74]
	DSS/rats	Reduces IL-23, IL-17, and NF-κB levels; raises PPAR-γ levels.	[75]
		Improves body weight and colon length; decreases C-reactive protein, TNF-α, IL-33, and IL-13 levels and MDA concentrations; elevates SOD levels and T-AOC.	[76]
		Improves the body weight; reduces the DAI and the levels of IL-1β, IL-6, TNF-α, CRP, PGE_2_, and MDA; increases the levels of SOD and IL-10 in both serum and colon tissue.	[77]

**Table 5:** Effects of various flavonols in different models of colitis.

Flavonol	Model	Properties	Ref.
Kaempferol	DSS/mice	Improves body weight and colon length; colitis was not improved in Bcrp−/− -Mrp2−/− mice.	[78]
		Down-regulates IL-1β, IL-6, TNF-α, iNOS, and COX-2 mRNA expression; decreases LTB4, PGE_2_, and NO plasma levels and MPO activity; up-regulates TFF3 mRNA colonic expression.	[79]
		Improves body weight and colon length; reduces the DAI; increases mRNA expression of IL-10, claudin-1, occludin, and ZO-1; decreases serum levels of TNF-α, IL-6, IL-1β, and LPS; suppresses mRNA expression of iNOS, MCP-1, COX-2, TNF-α, IL-6, IL-1β, NLRP3, p-NF-κB-P65, TLR4, and MyD88 in the colon, suggesting inhibition of the LPS–TLR4–NF-κB inflammatory pathway.	[80]
Myricetin		Increases TGF-β and IL-10 levels and the proportion of Treg cells; ameliorates the severity of inflammation in colitis.	[81]
		Improves colon length; reduces the DAI, IL-6 and IL-17 levels, expression of RORγt, and the proportion of Th17 cells; increases colonic IL-10 and IL-22 levels, expression of Foxp3, and the proportion of Treg cells; positively modulates the AhR signaling pathway; binds to AhR and promotes its activation.	[82]
		Improves body weight loss; decreases histology scores; reduces MDA, NO, and MPO production and IL-6 and IL-1β levels; increases GSH and SOD activities.	[83]
Myricitrin		Suppresses Akt/PI3K-dependent phosphorylation; decreases NF-κB, ERK1/2, MAPK p38, and c-JNK phosphorylation, TNF-α and IL-6 mRNA expression, and CXCL1/KC and IL-6 protein levels.	[84]
Galangin		Reduces colon length shortening, the DAI, MPO activity, and IL-6, IL-1β, and TNF-α levels; raises LC3B, ATG12, ATG7, and ATG5 protein expression; stimulates the formation of autophagosomes.	[85]
		Improves tissue damage and histopathological alterations; down-regulates TLR4 expression; inhibits NF-κB (p65) activation; decreases TNF-α and IL-6 levels; increases tissue GSH levels.	[86]
		Decreases IL-6 and TNF-α levels; raises IL-10 levels and SOD; reduces nitrites, TBARS, and MPO; inhibits p-Ikk-βα and p-NF-κB, Nrf2, iNOS, and COX-2 protein expression.	[87]
		Improves body weight and colon length; reduces the DAI and serum levels of IL-1, IL-6, and TNF-α; reduces expression of cleaved caspase-1 p20, ASC, and NLRP3 and MPO activity in the colon; inhibits HSP90; decreases p-AKT levels and oligomerization and stability of HSP90β.	[88]
Fisetin		Improves body weight and colon length; increases SOD level; reduces DAI, IL-1β, and TNF-α levels; activates the cGAS/STING signaling pathway.	[89]
		Decreases MPO activity, IL-6, IL-1β, and TNF-α production, and iNOS and COX-2 expression; inhibits the activation of NF-κB (p65) by suppressing IκBα phosphorylation and NF-κB (p65)–DNA binding activity; mitigates Akt and p38 phosphorylation; increases GSH; reduces MDA level.	[90]
Rutin		Improves body weight and colon length; reduces the DAI, levels of TNF-α, IL-1β, and IL-6, and MDA activity; increases CAT and SOD activities.	[91]
		Improves body weight and colon length; reduces the DAI and levels of IL-6, IL-18, TNF-α, and IL-1β in the colon and serum; decreases NLRP3 mRNA expression and protein levels of cleaved caspase-1, cleaved IL-1β, and NLRP3 in colonic tissue.	[92]
		Attenuates body weight loss, colon length shortening, the DAI, and goblet cell loss; reduces ICAM-1, HO-1, and iNOS mRNA levels, PI3K/Akt/GSK3β signaling pathway activation, and Bax protein; up-regulates IL-6, TNF-α, and IL-1β expression and the TNF-α/IL-10 ratio; modulates secretion levels and the ratios IL-5/IL-10, IL-6/IL-10, IL-1β/IL-10, TNF-α/IL-10, and IFN-γ/IL-10; regulates the MAPKs/NF-κB and p38/MK2 signaling pathways; raises Bcl-2 and PCNA expression and occludin, ZO-1, MUC3, and MUC2 mRNA expression; increases Treg cells; decreases CD4^+^ and CD8^+^ cells.	[93]
	Acetic acid/rats	Improves colon weight/colon length ratio; decreases serum levels of LDH and CRP; increases TAS; reduces TOS in colon; decreases both mRNA and protein expression of MYD88, TLR4, and HMGB1; reduces NF-kB expression in colon, suggesting inhibition of the HMGB1/TLR4/MYD88/NF-kB signaling pathway.	[94]

## Data Availability

No new data were created or analyzed in this study. Data sharing is not applicable to this article.
